# Mitochondrial phylogeography and population structure of the cattle tick *Rhipicephalus appendiculatus* in the African Great Lakes region

**DOI:** 10.1186/s13071-018-2904-7

**Published:** 2018-05-31

**Authors:** Gaston S. Amzati, Roger Pelle, Jean-Berckmans B. Muhigwa, Esther G. Kanduma, Appolinaire Djikeng, Maxime Madder, Nathalie Kirschvink, Tanguy Marcotty

**Affiliations:** 1 Unit of Integrated Veterinary Research, Department of Veterinary Medicine, Faculty of Sciences, Namur Research Institute for Life Sciences (NARILIS), University of Namur (UNamur), Rue de Bruxelles 61, 5000 Namur, Belgium; 2 Research Unit of Veterinary Epidemiology and Biostatistics, Faculty of Agricultural and Environmental Sciences, Université Evangélique en Afrique, P.O. Box 3323, Bukavu, Democratic Republic of the Congo; 3 Biosciences eastern and central Africa - International Livestock Research Institute (BecA-ILRI) hub, P.O. Box 30709-00100, Nairobi, Kenya; 4 Department of Biochemistry, School of Medicine, University of Nairobi, P.O. Box 30197-00100, Nairobi, Kenya; 5 Present address: Centre for Tropical Livestock Genetics and Health (CTLGH), The University of Edinburgh, Easter Bush, Midlothian, Scotland EH25 9RG UK; 6 Department of Veterinary Tropical Diseases, Faculty of Veterinary Science, University of Pretoria, P/Bag X04, Onderstepoort, 0110 South Africa

**Keywords:** East Coast fever, *Theileria parva*, *Rhipicephalus appendiculatus*, Phylogenetic, *cox*1, *12S* rRNA, Ticks, Evolutionary history, Agro-ecological zones, Population genetics

## Abstract

**Background:**

The ixodid tick *Rhipicephalus appendiculatus* is the main vector of *Theileria parva*, wich causes the highly fatal cattle disease East Coast fever (ECF) in sub-Saharan Africa. *Rhipicephalus appendiculatus* populations differ in their ecology, diapause behaviour and vector competence. Thus, their expansion in new areas may change the genetic structure and consequently affect the vector-pathogen system and disease outcomes. In this study we investigated the genetic distribution of *R. appendiculatus* across agro-ecological zones (AEZs) in the African Great Lakes region to better understand the epidemiology of ECF and elucidate *R. appendiculatus* evolutionary history and biogeographical colonization in Africa.

**Methods:**

Sequencing was performed on two mitochondrial genes (*cox*1 and *12S* rRNA) of 218 ticks collected from cattle across six AEZs along an altitudinal gradient in the Democratic Republic of Congo, Rwanda, Burundi and Tanzania. Phylogenetic relationships between tick populations were determined and evolutionary population dynamics models were assessed by mismach distribution.

**Results:**

Population genetic analysis yielded 22 *cox*1 and 9 *12S* haplotypes in a total of 209 and 126 nucleotide sequences, respectively. Phylogenetic algorithms grouped these haplotypes for both genes into two major clades (lineages A and B). We observed significant genetic variation segregating the two lineages and low structure among populations with high degree of migration. The observed high gene flow indicates population admixture between AEZs. However, reduced number of migrants was observed between lowlands and highlands. Mismatch analysis detected a signature of rapid demographic and range expansion of lineage A. The star-like pattern of isolated and published haplotypes indicates that the two lineages evolve independently and have been subjected to expansion across Africa.

**Conclusions:**

Two sympatric *R. appendiculatus* lineages occur in the Great Lakes region. Lineage A, the most diverse and ubiquitous, has experienced rapid population growth and range expansion in all AEZs probably through cattle movement, whereas lineage B, the less abundant, has probably established a founder population from recent colonization events and its occurrence decreases with altitude. These two lineages are sympatric in central and eastern Africa and allopatric in southern Africa. The observed colonization pattern may strongly affect the transmission system and may explain ECF endemic instability in the tick distribution fringes.

**Electronic supplementary material:**

The online version of this article (10.1186/s13071-018-2904-7) contains supplementary material, which is available to authorized users.

## Background

The ixodid brown ear tick *Rhipicephalus appendiculatus* is the main vector of the protozoan pathogen *Theileria parva*, the causative agent of a fatal lymphoproliferative cattle disease known as East Coast fever (ECF). East Coast fever is a highly pathogenic and the most economically important tick-borne disease of cattle in 12 sub-Saharan African countries, including Burundi, Democratic Republic of Congo and Rwanda [1]. *Rhipicephalus appendiculatus* is the most abundant tick in the Great Lakes region of Central Africa, where its burden and distribution vary significantly among agro-ecological zones (AEZs) [2–4]. The geographical dispersal pattern and population dynamics of this tick are mainly driven by climatic conditions, vegetation, host availability and mobility, grazing system and management practices [5, 6].

The Great Lakes region of Central Africa is characterised by cattle movement within and between countries for trade, breeding and pasture [7, 8]. During the pre-colonial period, immigrants originating from Rwanda and Burundi settled with their cattle in Congo in search of grazing lands. In addition, political unrest during the past three decades and rapidly increasing demand for animal products increased significantly the importation of live animals [9, 10]. This cross-border movement of cattle across geographical areas, together with bioclimatic conditions suitable for *R. appendiculatus*, could play a significant role in spreading ticks and pathogens [11–14]. Therefore, the spread and establishment of ticks from one geographical region to another might be setting up a complexity in the epidemiological status and control of the disease they transmit [15–17]. Thus, predicting vector-borne pathogen dynamics and emergence relies on better understanding of mechanisms underlying the genetic structure of their vectors [18, 19].

Ecological establishment and population genetic structure of ticks can be affected by founder events and gene flow, largely due to their dispersal across geographical zones through host migration [18, 20]. Arthropod vectors then respond differently to evolutionary forces such as migration, mutation, selection and genetic drift (promoted by bottlenecks) in their new environment [21, 22].

The adaptive mechanism of *R. appendiculatus* to changed environment suggests genetic divergence between geographical stocks and phenotypic variations, including diapause behaviour and vector competence to transmit *T. parva* [23, 24]. The degree to which different *R. appendiculatus* stocks acquire and transmit *T. parva* is partially genetically dependent because of the heritability of their susceptibility to infection [25]. Furthermore, there is a similar extensive genetic variation among *T. parva* strains in the field associated with different clinical features and disease outcomes, and variable cross-immunity levels. Studies suggest that there are also specific interactions between *R. appendiculatus* and *T. parva* stocks in the transmission dynamic system, significantly affecting the epidemiology of ECF [26]. Thus, phylogenetic and ecological analyses should provide useful information to control ECF by determining: (i) the genetic structure of *R. appendiculatus* populations; (ii) its dispersal pattern; and (iii) its demographic history [27, 28].

The genetic diversity of *R. appendiculatus* has been studied in different African countries using various molecular tools, such as mitochondrial DNA [14, 29] and micro- and minisatellite DNA markers [30]. Two distinct genetic groups have been described*,* namely the eastern and the southern African lineages [29]. More recently, Kanduma et al. [31] found that the two genetic groups are present in Kenya. These evidences show that *R. appendiculatus* genetic groups may have a wide geographical range, with different ecological preferences and phenology in sub-Saharan Africa [32–34], due to differences in body size [35] and diapause induction and intensity [36, 37].

Major gaps in knowledge still exist concerning the agro-ecological colonization and establishment of the *R. appendiculatus* lineages in the Great Lakes region of Central Africa, where cattle mobility seems to be the main factor of tick dispersal and epidemic instability of ECF [2, 38]. Thus, further studies on the population structure and phylogeography of *R. appendiculatus*, including ticks from distinct agro-ecological conditions of DR Congo, Burundi and Rwanda, are important to shed light on the intra and inter population variation, the dispersal pattern and the historical dynamics of the characterised lineages in various ecological situations of Africa. The objective of this study was to assess the evolutionary relationship between *R. appendiculatus* populations and to investigate the impact of geographical locations on its genetic structure, to better understand the epidemiology of ECF in the Great Lakes region. To achieve this objective, we sequenced and analysed fragments of the cytochrome *c* oxidase subunit 1 (*cox*1) and the *12S* ribosomal RNA (*12S* rRNA) gene loci. The genetic structure of *R. appendiculatus* provides clues to a better understanding of the epidemiology of ECF and insight into the development of targeted selective management and control strategies.

## Methods

### Study area

**Fig. 1:**
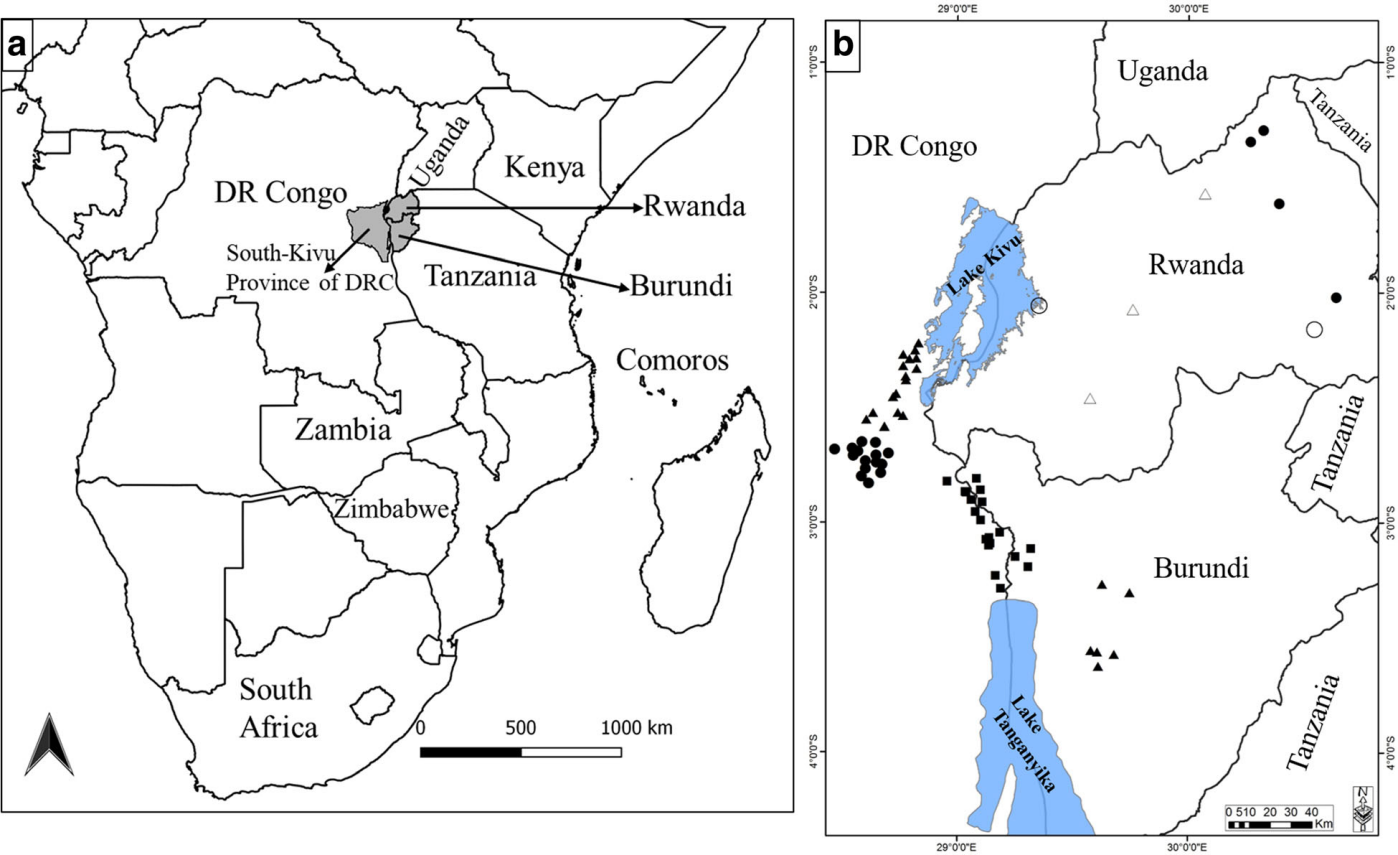
Sampling sites of *Rhipicephalus appendiculatus* ticks in DRC, Rwanda and Burundi. **a** Map of Africa showing the study area (in grey) and other countries where the tick was previously sequenced (indicated by their names). **b** Sampling localities of *R. appendiculatus* and their altitudes (squares: AEZ1 altitude < 1200 m; circles: AEZ2 altitude 1200–1600 m; and triangles: AEZ3 altitude > 1600 m). The sites represented by empty circles and triangles  show sampling locations described by Mtambo et al. [29]

Ticks were collected from cattle in six agro-ecological zones (AEZs) of the Central African Great Lakes region (Democratic Republic of Congo, Rwanda and Burundi) (Fig. 1). The three countries share the Ruzizi Valley, which consists of lowlands along the Ruzizi River flowing between Lake Kivu and Lake Tanganyika. A summary description of the characteristics of the six AEZs is presented in Table 1. Briefly, although the study area is close to the equator, the wide range of altitudes (700 to 3000 m above sea level) mitigates significantly the tropical climate attributes and therefore the AEZs limits. Generally, the rainfall period is long and bimodal (with variations in the duration between AEZs). An early rainy season starts approximately in September and ends in December, while the late rainy season lasts from February to May, followed by a dry season from June to August. There is an intervening short dry period between the two rainy seasons of about 15 days in January and/or February depending on the AEZs. Rainfall and temperature are strongly influenced by altitude ranges. Rainfall increases while temperature decreases with increasing elevation. The average temperature ranges between 12–25 °C and the annual average rainfall from 800–1100 mm in the Ruzizi Valley to 1300–2000 mm in the highlands.

**Table 1:** Geographical and climatic attributes of the six agro-ecological zones (AEZ)

Country	Agro-ecological zone (AEZ)	Altitude (m)	Temperature (°C)	Rainfall (mm/year)	Rainy season	Sample size (no. of ticks)
DRC	AEZ1	780–1100	23–25	800–1000	October-April	46
	AEZ2	1200–1600	17–21	1000–1500	September-May	54
	AEZ3	1600–2800	12–19	1350–2000	September-May	46
Burundi	AEZ1	774–1100	23–24	800–1100	November-May	26
	AEZ3	1700–2800	14–15	1300–2000	September-May	17
Rwanda^a^	AEZ2	1200–1500	21–24	800–950	November-May	20

*Notes*: DRC (AEZ1: Lowlands, AEZ2: Midlands, AEZ3: Highlands); Burundi (AEZ1: Lowlands, AEZ3: Highlands); Rwanda (AEZ2: eastern low plateau which is the lowlands of Rwanda as described by Bazarusanga et al. [3])

^a^Sequences previously described in Mtambo at al. [29] are not included in the 20 samples from Rwanda and thus were not used in the population genetic analysis presented in Tables 2, 3 and 4

In eastern DRC, ticks were collected along an altitudinal transect in South-Kivu province. Based on elevation and geographical position within the transect, three major AEZs were defined from lowlands to highlands, namely, DRC AEZ1 (lowlands) in the Ruzizi Valley, DRC AEZ2 (midlands) in the administrative district of Walungu and DRC AEZ3 (highlands) in the district of Kabare and the highlands part of Walungu (Mulumemunene). The lowlands region (DRC AEZ1) is the warmest AEZ, characterised by a tropical dry climate (semi-arid), with a cool dry season of 4–5 months. Cattle are raised generally in an open grazing system in communal pastures of savannah grassland. In contrast, the “upland Kivu” (DRC AEZ2 and AEZ3) has a montane humid tropical climate and is much cooler. The warm dry season lasts for 3–4 months, with occasional and poorly distributed rainfall.

Ticks from Burundi were collected from two AEZs. The sampled administrative districts included Rugombo and Gihanga in the Imbo lowlands (Burundi AEZ1) and Muramvya, Mwaro and Mugamba districts of the Congo-Nile highlands (Burundi AEZ3). The Imbo region extends along the Ruzizi River and the North of Lake Tanganyika. The vegetation is composed of savannah and small patches of forest.

Ticks from Rwanda were obtained from the eastern low plateau in Nyagatare, Gatsibo and Kayonza districts (Rwanda AEZ2). This region includes the savannah of eastern province of Rwanda. The eastern semi-arid plateau zone is the most important pastoral region in Rwanda, holding 40% of the national herd raised in a communal grazing system. The vegetation type is largely savannah and some river bank woods.

### Tick sampling and morphological identification

Ticks were collected from cattle during a cross-sectional survey conducted from February to April 2015 (late rainy season) in the six AEZs. In each AEZ, 8 to 12 cattle herds were selected randomly using a random number generator in Microsoft Excel. From ticks collected in each herd representing a location or village, 4 to 5 ticks were selected using the same random process for further analysis. The number of ticks sampled in each population is shown in Table 1. All ticks were directly immersed in 70% ethanol for preservation and morphologically identified using the identification key of Walker et al. [39]. Morphological identification was confirmed at the tick unit at the International Livestock Research Institute (ILRI, Kenya). Ten additional specimens originating from Simanjiro district in northern Tanzania were obtained from the Sokoine University of Agriculture in Tanzania.

### DNA extraction, PCR amplification and sequencing

Total genomic DNA was isolated from whole individual ticks using the DNeasy® Blood and Tissue Kit (Qiagen GmbH, Hilden, Germany) according to the standard manufacturer’s protocol, except that an additional incubation of 10 min at 56 °C was added after mixing the sample with 200 μl buffer AL to ensure optimal cell lysis. Prior to extract DNA, ticks were washed twice in double distilled water and left to dry for 10 min at room temperature and homogenized by grinding.

Given the suitability of mitochondrial genes to discriminate intraspecific variation in ticks [29, 31], we amplified the *cox*1 and *12S* rRNA gene loci to assess the genetic diversity and phylogenetic relationships of *R. appendiculatus* populations*.* A 710 bp fragment of *cox*1 gene locus was amplified with the forward primer LCO1490 (5'-GGT CAA CAA ATC ATA AAG ATA TTG G-3') and the reverse primer HC02198 (5'-TAA ACT TCA GGG TGA CCA AAA AAT CA-3') as previously described by Folmer et al. [40]; for the *12S* rRNA gene locus we used the primers SR-J-1499 (5'-TAC TAT GTT ACG ACT TAT-3') and SR-N-14594 (5'-AAA CTA GGA TTA GAT ACC C-3') with a fragment size of 380 bp, described in Simon et al. [41]. PCR amplifications of both *cox*1 and *12S* rRNA gene fragments were performed using 50 ng of genomic DNA, 25 μl of 2× AccuPower® PCR PreMix (Bioneer PCR-PreMix, Seoul, South Korea), 0.2 μM each of forward and reverse primers, and nuclease free water added up to a final reaction volume of 50 μl. The thermal cycling program for *cox*1 consisted of an initial denaturation at 95 °C for 3 min followed by 35 cycles of denaturation at 94 °C for 1 min, annealing at 40 °C for 1 min and extension at 72 °C for 1 min. The final extension was carried out for 10 min at 72 °C. PCR parameters of *12S* rRNA gene fragment were as described for *cox*1, except that the annealing temperature was 52 °C and the extension time was 90 s. PCR products were analysed by electrophoresis on a 1.8% agarose gel. Amplicons were purified using the QIAquick® PCR Purification Kit (Qiagen GmbH, Hilden, Germany) following the manufacturer’s instructions. Both strands were sequenced directly with the same primers used for PCR, on an ABI 3730 capillary sequencer (Applied Biosystems, California, USA).

### Sequences editing, blasting and multiple alignments

Forward and reverse chromatograms for each individual tick were visually checked. Sequences were manually edited and assembled using CLC Main Workbench software v7.8.1 (CLC Bio, Aarhus, Denmark). Multiple sequences were aligned with CrustalW algorithm using default settings in the same software. The sequences were then trimmed to exclude poor quality bases and obtain uniform sizes. The final sequence sizes were 586 bp and 346 bp for *cox*1 and *12S* rRNA genes, respectively. Aligned and trimmed sequences were subjected to a BLAST search against all NCBI nucleotide databases, with default settings to confirm their species identity. A total of 219 sequences for *cox*1 and 126 for *12S* were obtained after quality check processing. To check for amplification of nuclear pseudogenes and confirm protein coding, all *cox*1 nucleotide sequences were translated into their amino acid sequences to examine the presence of ambiguous stop codons for correct coding of invertebrate mitochondrial DNA.

### Genetic diversity and population genetic structure

Multiple sequences extracted from the alignments were used to construct haplotypes in DnaSP software v5.10.01 [42]. Genetic variation within populations was estimated on *cox*1 gene sequences. Population genetic indices represented by number of haplotypes (*h*), number of segregating sites (*S*), haplotype diversity (*Hd*), mean number of pairwise nucleotide differences within population (*K*) and nucleotide diversity (*π*) were calculated for each AEZs (named populations) and for the overall data set using Arlequin v3.5.2.2 [43] and DnaSP. The population genetic structure among AEZs and among haplogroups was evaluated by an analysis of molecular variance (AMOVA) performed in Arlequin. The significance of AMOVA fixation indices was evaluated based on 1,023 random permutations. In addition, to assess the level of genetic distance/differentiation between populations, we estimated gene flow expressed as of the absolute number of migrants (*Nm*) exchanged among populations, average number of nucleotide differences between populations (*K*_*XY*_) and population pairwise genetic differentiation (*F*_*ST*_) also in Arlequin [44]. Their significance was tested using 1000 random permutations. The genetic differentiation and population structure statistics were tested under the hypothesis that different populations are represented by distinct genetic groups or are exchanging migrants. These analyses were performed for combined data (of the main haplogroups identified) to understand the effect of coexistence of the haplogroups in the genetic structure and diversity.

### Demographic and spatial expansion history analyses

The historical population dynamics and structure was inferred from mismatch distribution of *cox1* haplotypes implemented in Arlequin [45], which compared the observed distribution of pairwise nucleotide differences between haplotypes and that expected under a population expansion model for each population, haplogroup and overall data. Multimodal mismatch pattern is assumed to define a population of demographic equilibrium or constant size, whereas sudden or expanding population is characterised by unimodal distribution. The sum of square deviation (*SSD*) were estimated to determine if the observed mismatch deviated significantly from a population expansion model, and the Harpending’s raggedness index (*RI*) were used to evaluate the smoothness of mismatch distribution [46]. In addition, to detect deviation from neutrality expectations or mutation-drift equilibrium we performed analysis of Fu’s *Fs* [47] and Tajima’s *D* [48] statistics in Arlequin and DnaSP. Tajima’s *D* test is based on the difference between the number of polymorphic sites and the mean number of nucleotide pairwise differences, while Fu’s *Fs* test is based on haplotype frequencies. The significance of all these statistics was tested by bootstrap resampling of 1000 coalescent simulations. Significant negative values of neutrality statistics should indicate a signature of historical event of population expansion, whereas significantly positive values indicate events such as recent population bottleneck, population subdivision or presence of some recent immigrants in a population. Values near zero and not significant, indicate that population size is constant.

### Phylogenetic and phylogeographical reconstruction

Different published haplotype sequences of *R. appendiculatus* from South Africa, Kenya, Grande Comore, Zambia, Zimbabwe, Uganda and Rwanda were retrieved from the National Center for Biotechnology Information (NCBI) database (see Additional file 1: Table S1) and were compared with sequences obtained in the present study. Phylogenetic reconstruction was performed separately on *cox*1 and *12S* rRNA gene sequences to determine the relationship among populations and possible historical dispersal event. To find the evolutionary substitution model that best describe the evolution of *cox*1 and *12S* rRNA gene sequences, we performed a hierarchical likelihood ratio test, based on the lowest Bayesian information criterion using MEGA v7.0 [49]. The nucleotide substitution model selected was then used to perform a Neighbor-Joining (NJ) and/or Maximum Likelihood (ML) algorithm in MEGA. The stability and branches support were obtained using 1000 bootstrap permutations. *Rhipicephalus eversti* and *Rhipicephalus microplus* from this study (GenBank accession numbers MF458972 and MF458973 for *cox*1 and MF479198 and MF479199 for *12S* RNA genes) and *Rhipicephalus turanicus* obtained from the GenBank (accession numbers KU880574 and DQ849231 for *cox*1 and *12S* rRNA genes, respectively) were used as outgroup taxa. A Median-Joining (MJ) network was constructed to investigate the phylogenetic and ancestral relationship among haplotypes using PopArt Software [50].

## Results

### Morphological and molecular identification of ticks

PCR fragments of the mitochondrial *cox*1 and *12S* rRNA gene loci were successfully generated from 219 and 126 individual ticks, respectively, originating from the three AEZs of DRC, the two AEZs of Burundi, one AEZ of Rwanda and specimens from Tanzania. The 10 sequences from Tanzania and the sequences previously described in Rwanda were not included in the population genetic and structure analyses. Generated nucleotide sequences were aligned with the reference haplotype sequences retrieved from the GenBank (Additional file 1: Table S1). The final fragment length obtained for *cox*1 was 586 bp and *12S* rRNA yielded a fragment of 346 bp long, with no indels detected in both genes. Their morphological identification has been confirmed by the high molecular identity (99–100%) with known sequences of *R. appendiculatus* found in GenBank (Additional file 2: Table S2). Haplotype sequences (55 and 23 sequences for *cox*1 and *12S* rRNA, respectively) obtained for each of the six AEZs were deposited and are available in GenBank (GenBank accession numbers: MF458895-MF458949 and MF479166-MF479188 for *cox*1 and *12S* rRNA genes, respectively).

### Phylogenetic relationships and haplotypes distribution

**Fig. 2:**
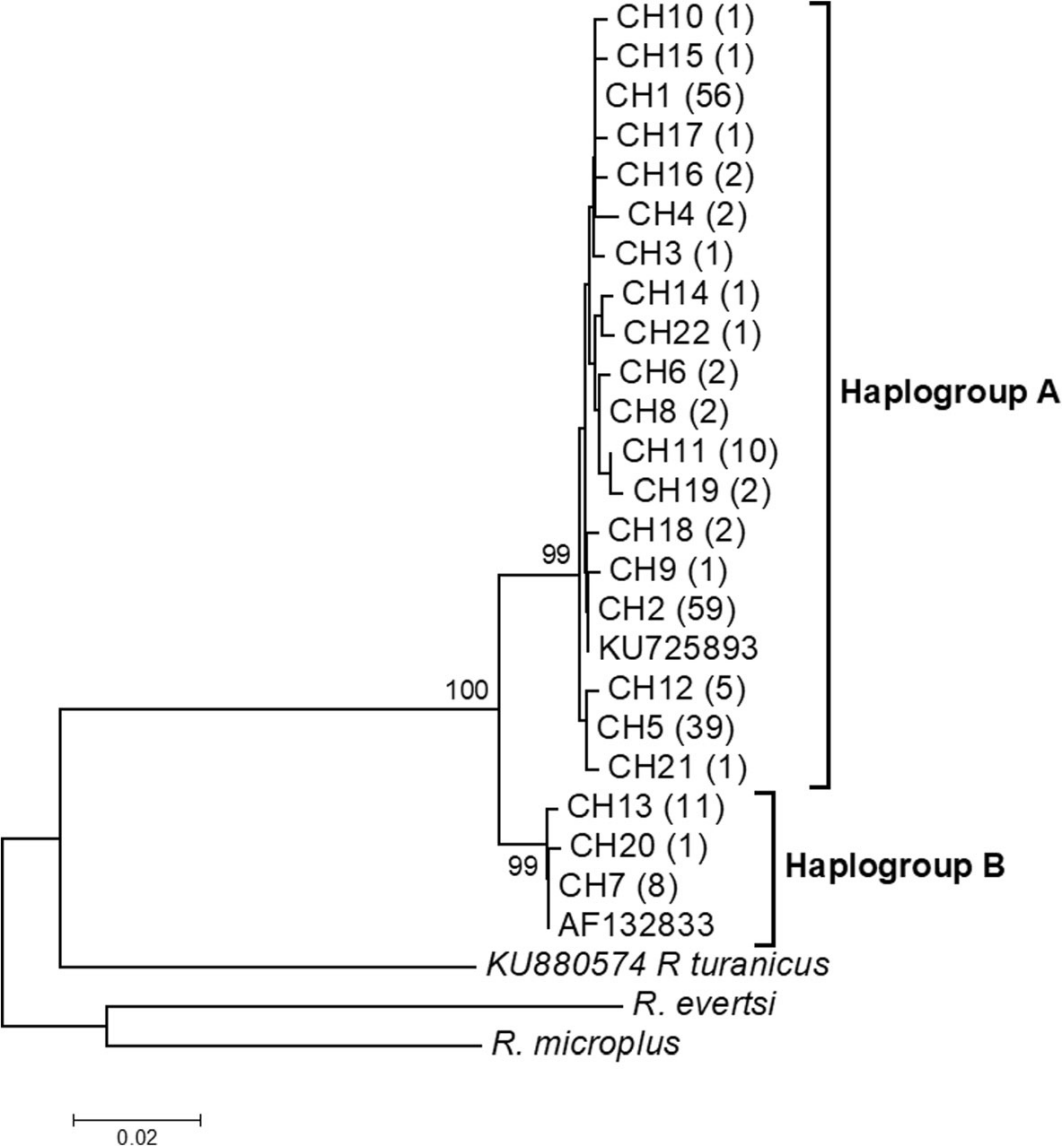
Phylogenetic tree of *R. appendiculatus cox*1 haplotypes*.* The evolutionary history was inferred by using the neighbor-joining method based on the Tamura 3-parameter model. A discrete Gamma distribution was used to model evolutionary rate differences among sites. Bootstrap values (> 80) are displayed above nodes. CH1-22 are haplotype names. The values in parentheses correspond to the frequency of each haplotype. KU725893 and AF132833 are GenBank accession numbers for *R. appendiculatus* sequences used as reference haplotypes from Kenya and Zimbabwe, respectively. Two species (*R. eversti* and *R. microplus*) obtained in this study and *R. turanicus* from GenBank (accession number: KU880574) were included as outgroups

**Fig. 3:**
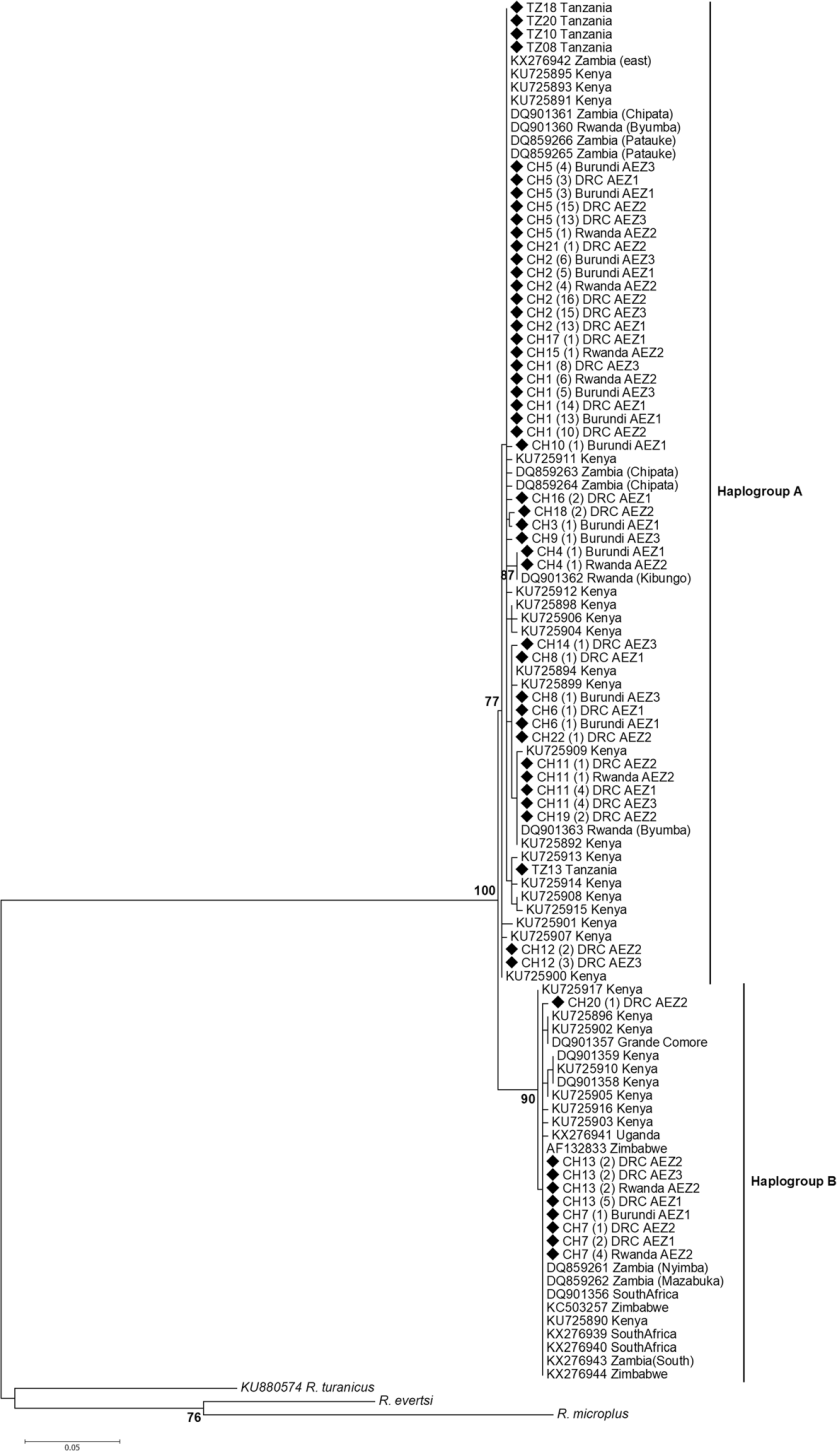
Phylogenetic tree of *cox*1 haplotypes displaying the relationship between the *R. appendiculatus* specimens in sub-Saharan African countries*.* The evolutionary history was inferred by using the maximum likelihood method based on the Tamura 3-parameter model. A discrete Gamma distribution was used to model evolutionary rate differences among sites [5 categories (+G, parameter = 0.39)]. The tree is drawn to scale, with branch lengths measured in the number of substitutions per site. Bootstrap scores > 70 are displayed to support nodes. The values in bracket behind haplotype names correspond to the frequency of each haplotype. Haplotype sequences (CH1-22) obtained in the present study are indicated by a black square. *Rhipicephalus eversti* and *R. microplus* obtained in this study and *R. turanicus* (GenBank number: KU880574) were used as outgroups

**Fig. 4:**
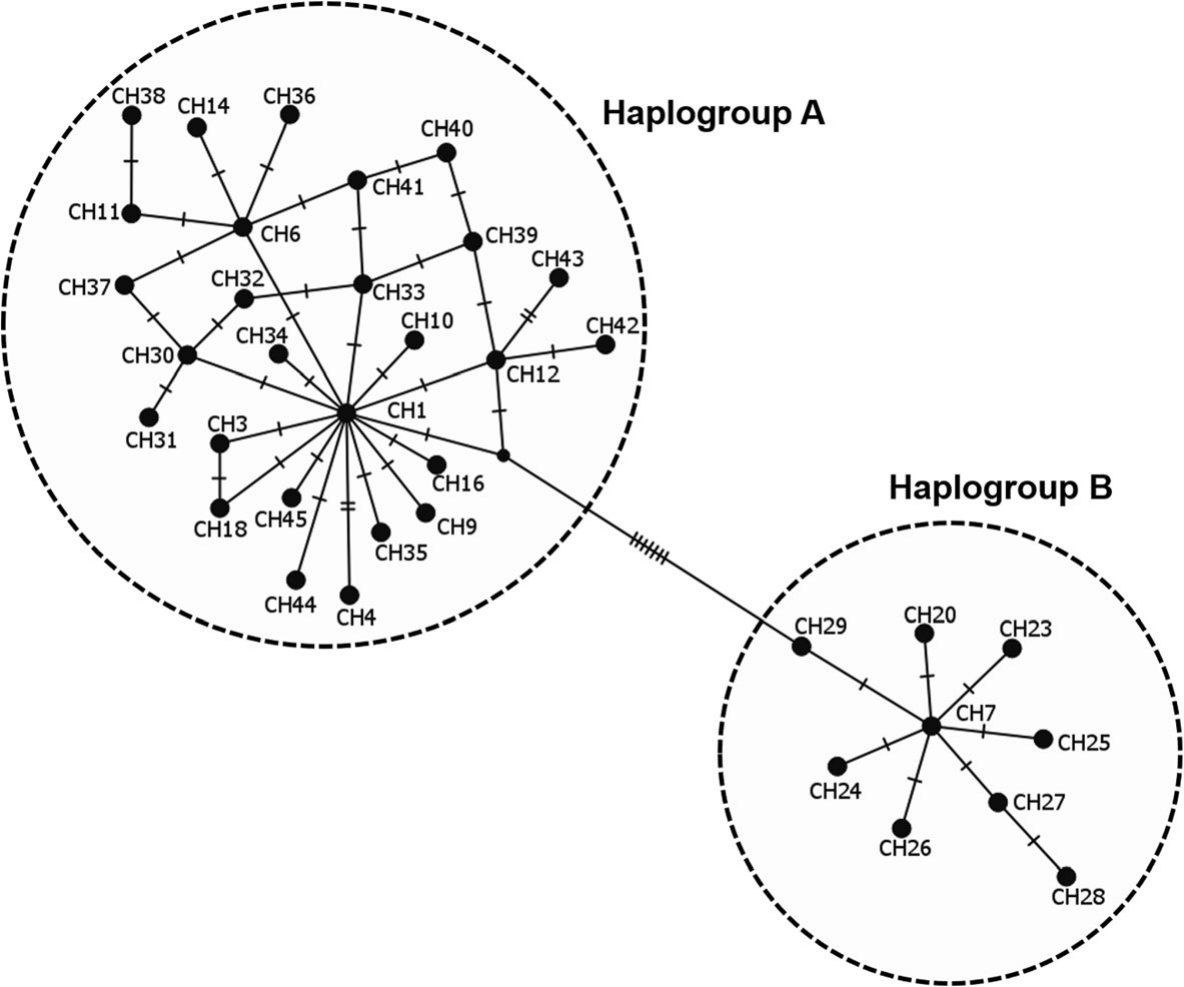
Median-joining network of the 36 *cox*1 haplotypes for *R. appendiculatus* ticks across sub-Saharan African countries. Lines represent mutations and the dot corresponds to a possible intermediate haplotype. Each circle denotes a unique haplotype. Haplotype frequencies are not shown here, but their occurrences across Africa are presented in Table 5

The overall sequence analysis revealed that *cox*1 had 27 polymorphic sites, 21 of which were parsimony informative and 6 were singletons, yielding 22 haplotypes (Additional file 3: Table S3). *cox*1 amino acid sequences did not contain any internal stop codon or indel. Most nucleotide mutations were synonymous, except one non-synonymous change identified at position 32 of the haplotype CH22 (change from an Alanine to a Threonine). The highest number of *cox*1 haplotypes was found in DRC AEZ1, while the lowest was observed in Burundi AEZ3 (Table 2). The *12S* rRNA gene provided 10 polymorphic sites, five of which were parsimony informative, defining 9 haplotypes. The 22 *cox*1 haplotype sequences obtained in this study were submitted to GenBank under accession numbers MF458950-MF458971 and the nine *12S* rRNA gene haplotypes were deposited under accession numbers MF479189-MF479197. The phylogenetic relationships among *cox*1 haplotypes inferred by a NJ phylogenetic tree (Fig. 2), a ML tree (Fig. 3) and a MJ network (Fig. 4) produced identical topologies and detected two distinct clades or lineages strongly-supported by a NJ bootstrap value of 100%. The two lineages diverged at least by 12 mutational steps (Additional file 3: Table S3) but shared a wide range of agro-ecological and geographical conditions in the Great Lakes region. The first lineage, named “haplogroup A”, was represented by the most frequent haplotypes (CH1, CH2 and CH5) and comprised in total 19 haplotypes consisting of 189 of the 209 sequences analysed (90%), whereas the second lineage “haplogroup B” included three haplotypes (CH7, CH13, CH20) and had a total of 20 sequences (10%) (Fig. 2, Additional file 3: Table S3). Haplogroup B comprised of haplotypes present in most AEZs except the highlands region of Burundi (Burundi AEZ3). These *cox*1 haplogroups presented a star-like pattern in the MJ network with less frequent and single haplotypes connected together to the predominant or ancestral haplotypes generally by single substitutions (Fig. 4), supporting the hypothesis of a recent population expansion. The phylogenetic relationships found in the *cox*1 gene were fully congruent with those revealed by the ML tree performed on *12S* rRNA haplotypes.

**Table 2:** *Rhipicephalus appendiculatus cox*1 haplotypes distribution (%) and genetic diversity indices in six agro-ecological zones of the Democratic Republic of Congo, Burundi and Rwanda

Country	AEZ	*n*	h	Haplotype (frequency in %)^a,b^	Haplogroup^c^ (%)		Genetic diversity indices			
					A	B	S (PIS)	*Hd* (SD)	*K*	π (SD)
DRC	AEZ1	46	10	**CH1(30), CH2(28), CH5(6),** CH6(2), CH7(4), CH8(2), CH11(9), CH13(11), CH16(4), CH17(2)	85	15	17 (16)	0.82 (0.03)	4.4 (2.2)	0.007 (0.002)
	AEZ2	54	12	**CH1(18), CH2(30)**, **CH5(28)**, CH7(2), CH11(2), CH12(4), CH13(4), CH18(4), CH19(4), CH20(2), CH21(2), CH22(2)	92	8	20 (17)	0.81 (0.03)	3.1 (1.6)	0.005 (0.001)
	AEZ3	46	7	**CH1(17), CH2(33), CH5(28),** CH11(9), CH12(6), CH13(4), CH14(2)	96	4	16 (15)	0.79 (0.03)	2.4 (1.3)	0.004 (0.001)
Burundi	AEZ1	26	8	**CH1(50), CH2(19)**, CH3(4), CH4(4), **CH5(11)**, CH6(4), CH7(4), CH10(4)	96	4	18 (3)	0.72 (0.08)	2.01 (1.2)	0.003 (0.001)
	AEZ3	17	5	**CH1(29), CH2(35), CH5(23)**, CH8(6), CH9(6)	100	0	4 (2)	0.77 (0.06)	1.1 (0.9)	0.002 (0.0003)
Rwanda	AEZ2	20	8	**CH1(30), CH2(20)**, CH4(5), **CH5(5)**, CH7(20), CH11(5), CH13(10), CH15(5)	70	30	18 (13)	0.85 (0.05)	6.3 (3.1)	0.011 (0.002)
Total		209	22	**–**	90	10	27 (21)	0.81(0.01)	3.4 (1.7)	0.006 (0.0006)

*Abbreviations*: *AEZ* agro-ecological zones, *n* number of sequences, h number of haplotypes, *S* segregation sites, *PIS* parsimony informative sites, *Hd* haplotype diversity, *SD* standard deviation; *K* mean number of pairwise nucleotide differences, π nucleotide diversity, *CH1-22* names of *cox*1 haplotypes

^a^Haplotypes belonging to the haplogroup B are underlined

^b^Bold indicates shared haplotypes by all agro-ecological zones

^c^A and B are haplogroup names

The distribution of haplotypes presented in Table 2 showed that there were shared and private *cox*1 haplotypes confined to restricted AEZs. Three haplotypes CH1 (27%), CH2 (28%) and CH5 (19%) were shared between all AEZs and were by far the most ubiquitous in the region, accounting for 74% (154 sequences) of the overall dataset (Additional file 3: Table S3). Haplotype CH1 was detected in 13 (50%) of the 26 sequences from lowlands of Burundi (Burundi AEZ1). Two haplotypes (CH11 and CH13) defined by 10 and 11 sequences, respectively, were common in all the AEZs of DRC and in Rwanda AEZ2. Haplotype CH12 was exclusive to DRC AEZ2 and AEZ3. Nine out of the 22 haplotypes were found in single individuals; therefore, they belonged to particular AEZs (Additional file 3: Table S3). Furthermore, two *12S* rRNA haplotypes (12SH1 and 12SH2) were the most abundant, representing 90% of the 126 sequences analysed. Haplotypes 12SH4 and 12SH5 had together 8 (6%) out of the 126 analysed sequences. The presence of single haplotypes indicates high frequency of rare alleles, which suggests a recent population expansion.

### Population genetic diversity

Population genetic indices were estimated using *cox*1 nucleotide sequences and are shown in Table 2. The overall haplotype diversity (*Hd*) and nucleotide diversity (π) were 0.81 and 0.006, respectively. The haplotype diversity ranged from 0.72 in Burundi AEZ1 to 0.85 in Rwanda AEZ2 and the nucleotide diversity (π) values ranged from 0.002 in Burundi AEZ3 to 0.011 in Rwanda AEZ2. The average number of nucleotide pairwise differences (k) was 3.4 for the overall dataset, with the highest value observed in Rwanda AEZ2 (6.3). Altogether, these population genetic indices showed that the diversity of *R. appendiculatus* is quite different among AEZs. Ticks from Rwanda AEZ2 were more highly diverse than those from three AEZs of DRC and the two AEZs of Burundi. We also observed an excess of singleton mutations in Burundi AEZ1 (15 out of 18 polymorphic sites), suggesting a sudden population expansion. These data analysed separately by haplogroups (Table 3), showed that the differences of genetic diversity among AEZs was largely affected by the frequency distribution of the two *cox*1 haplogroups (Table 2).

**Table 3:** Genetic diversity and evolutionary dynamics of the two haplogroups (A and B) identified from *cox*1 sequences of *R. appendiculatus*

Genetic indices and statistics	Haplogroup A							Haplogroup B	Overall data set
	DRC			Burundi		Rwanda AEZ2	Haplogroup A (overall)		
	AEZ1	AEZ2	AEZ3	AEZ1	AEZ3				
Diversity indices									
Number of sequences	39	50	44	25	17	14	189	20	209
Number of polymorphic sites	7	9	6	8	4	7	18	2	27
Number of haplotypes	8	9	6	7	5	6	19	3	22
Haplotype diversity (SD)	0.76 (0.044)	0.78 (0.032)	0.77 (0.033)	0.7 (0.084)	0.77 (0.057)	0.77 (0.089)	0.77 (0.016)	0.56 (0.063)	0.81 (0.01)
Nucleotide diversity (SD)	0.002 (0.0003)	0.0025 (0.0003)	0.002 (0.0003)	0.002 (0.0004)	0.002 (0.0003)	0.002 (0.0006)	0.002 (0.0001)	0.001 (0.0002)	0.006 (0.0006)
Neutrality tests									
Tajima's *D* (*P*-value)	-0.6 (0.35)	-0.79 (0.23)	0.096 (0.6)	-1.4 (0.049)*	-0.21 (0.45)	-1.4 (0.07)	-1.5 (0.032)*	0.24 (0.67)	-0.93 (0.23)
Fu's *Fs* (*P*-value)	-2.4 (0.08)	-2.5 (0.097)	-0.1 (0.49)	-2.6 (0.027)*	-1.1 (0.18)	-2.1 (0.041)*	-10.4 (0.001)*	0.2 (0.49)	-3.8 (0.15)
Demographic expansion									
Sum of Squared deviation (*P*-value)	0.002 (0.66)	0.004 (0.31)	0.002 (0.49)	0.002 (0.72)	0.02 (0.17)	0.001 (0.9)	0.0008 (0.44)	0.029 (0.049)*	0.017 (0.1)
Harpending’s Raggedness index (*P*-value)	0.06 (0.39)	0.079 (0.21)	0.06 (0.38)	0.05 (0.71)	0.16 (0.12)	0.06 (0.73)	0.057 (0.2)	0.21 (0.1)	0.049 (0.51)
Spatial expansion									
Sum of Squared deviation (*P*-value)	0.002 (0.5)	0.004 (0.21)	0.002 (0.4)	0.002 (0.67)	0.02 (0.1)	0.001 (0.9)	0.0008 (0.26)	0.029 (0.011)*	0.036 (0.28)
Harpending’s Raggedness index (*P*-value)	0.064 (0.39)	0.079 (0.21)	0.062 (0.43)	0.05 (0.75)	0.16 (0.12)	0.06 (0.72)	0.057 (0.22)	0.21 (0.12)	0.049 (0.74)

*Abbreviations*: *D* Tajima’s neutrality statistic, *Fs* Fu’s neutrality statistic

*Values are statistically significant at *P* < 0.05; significance was determined using 1000 coalescent simulations

### Population structure and ecological differentiation based on *cox*1 haplotypes

Analysis of molecular variance (AMOVA) based on *cox*1 sequences revealed statistically significant variance among the six AEZs analysed together for both combined haplogroups and haplogroup A alone (6%, *P* < 0.001) (Additional file 4: Table S4). The genetic variability among individuals within AEZs explained the large majority of molecular variance (94%) for the overall dataset, suggesting an admixture between different populations and the coexistence of the two genetically divergent lineages (Fig. 2, Table 2). This is consistent with moderate genetic structure of *R. appendiculatus* in the Great Lakes region. The population differentiation indices were then calculated to compare the genetic variation observed among AEZs (Table 4). Both differentiation statistics based on pairwise estimates of the Inter-population nucleotide differences (*K*_*XY*_), the pairwise genetic distance (*F*_*ST*_), and the number of migrants (*Nm*) showed evidence of differentiation between some *R. appendiculatus* populations. The population pairwise genetic distance (*F*_*ST*_) varied from a negative and not significant value (*F*_*ST*_ = -0.014, *P* = 0.87) with infinite value of *Nm* (between DRC AEZ2 and AEZ3) to the strongest differentiation (*F*_*ST*_ = 0.19, *P* = 0.005) with low number of migrants (*Nm* = 2.1) between Burundi AEZ3 and Rwanda AEZ2. Pairwise *F*_*ST*_ values were found to be low and not significant between DRC AEZ3 and Burundi AEZ3 with infinite *Nm* and between DRC AEZ2 and Burundi AEZ3 associated to a high number of migrants (*Nm* = 11,875), indicating an excess of gene flow between these zones. DRC AEZ1 did not differ significantly with Burundi AEZ1 (*F*_*ST*_ = 0.022, *P* = 0.19) and Rwanda AEZ2 (*F*_*ST*_ = 0.018, *P* = 0.24). Tick populations from Rwanda AEZ2 showed a strong genetic differentiation with those from DRC AEZ3 (*F*_*ST*_ = 0.18, *P* < 0.001) and DRC AEZ2 (*F*_*ST*_ = 0.14, *P* = 0.03), indicating reduced or lack of gene flow between these populations. These results were confirmed by inter-population nucleotide differences (*K*_*XY*_), which was significant when populations were significantly differentiated by the pairwise *F*_*ST*_ statistic. Tick populations were compared by analysing haplogroup A sequences alone. The findings showed that ticks from Rwanda AEZ2 and the two AEZs of Burundi were not genetically different. They shared more migrants belonging to haplogroup A (Table 4). In general, the population differentiation was observed between lowlands and highlands AEZs.

**Table 4:** Population genetic statistics for pairwise comparison of different populations of *R. appendiculatus* from sequences of *cox*1 gene. Values in parentheses represent the *P*-value statistics

Population 1	Population 2	Haplogroup A and B			Haplogroup A		
		*K*_*XY*_	*F*_*ST*_	*Nm*	*K*_*XY*_	*F*_*ST*_	*Nm*
DRC AEZ1	DRC AEZ2	3.8 (0.044)*	0.036 (0.044)*	13.2	1.5 (< 0.001)*	0.11 (< 0.001)*	4
DRC AEZ1	DRC AEZ3	3.6 (0.032)*	0.057 (0.032)*	8.3	1.5 (0.001)*	0.1 (0.001)*	4.3
DRC AEZ1	Burundi AEZ1	3.3 (0.15)	0.022 (0.19)	22.2	1.2 (0.31)	0.002 (0.33)	243.6
DRC AEZ1	Burundi AEZ3	3 (0.059)	0.056 (0.087)	8.4	1.3 (0.087)	0.043 (0.082)	11.2
DRC AEZ1	Rwanda AEZ2	5.4 (0.3)	0.018 (0.24)	26.8	1.3 (0.78)	-0.026 (0.79)	∞
DRC AEZ2	DRC AEZ3	2.7 (0.85)	-0.014 (0.87)	∞	1.4 (0.93)	-0.016 (0.93)	∞
DRC AEZ2	Burundi AEZ1	2.7 (0.003)*	0.060 (0.011)*	7.9	1.5(< 0.001)*	0.13 (< 0.001)*	3.2
DRC AEZ2	Burundi AEZ3	2.1 (0.23)	0.00004 (0.43)	11876	1.3 (0.46)	-0.012 (0.54)	∞
DRC AEZ2	Rwanda AEZ2	5.2 (0.003)*	0.14 (0.003)*	3.1	1.6 (0.006)*	0.11 (0.004)*	3.8
DRC AEZ3	Burundi AEZ1	2.4 (< 0.001)*	0.076 (0.006)*	6.1	1.5(< 0.001)*	0.14 (< 0.001)*	3.2
DRC AEZ3	Burundi AEZ3	1.8 (0.32)	-0.005 (0.43)	∞	1.3 (0.39)	-0.009 (0.46)	∞
DRC AEZ3	Rwanda AEZ2	5.1 (< 0.001)*	0.18 (< 0.001)*	2.2	1.6 (0.009)*	0.11 (0.011)*	3.9
Burundi AEZ1	Burundi AEZ3	1.6 (0.074)	0.034 (0.08)	12.2	1.2 (0.077)	0.063 (0.073)	7.4
Burundi AEZ1	Rwanda AEZ2	4.8 (0.030)*	0.14 (0.03)*	3.1	1.2 (0.97)	-0.039 (0.98)	∞
Burundi AEZ3	Rwanda AEZ2	4.6 (0.005)*	0.19 (0.005)*	2.1	1.3 (0.16)	0.044 (0.14)	10.9

*Abbreviations*: *K*_*XY*_ average number of nucleotide differences between populations, *F*_*ST*_ pairwise genetic distance *F*-statistic based on nucleotide sequences (Right’s fixation index), *Nm* number of migrants between populations

*Values are statistically significant at *P* < 0.05; significance was determined using 1000 coalescent simulations.

### Demographic and dispersal dynamics of *R. appendiculatus*

**Fig. 5:**
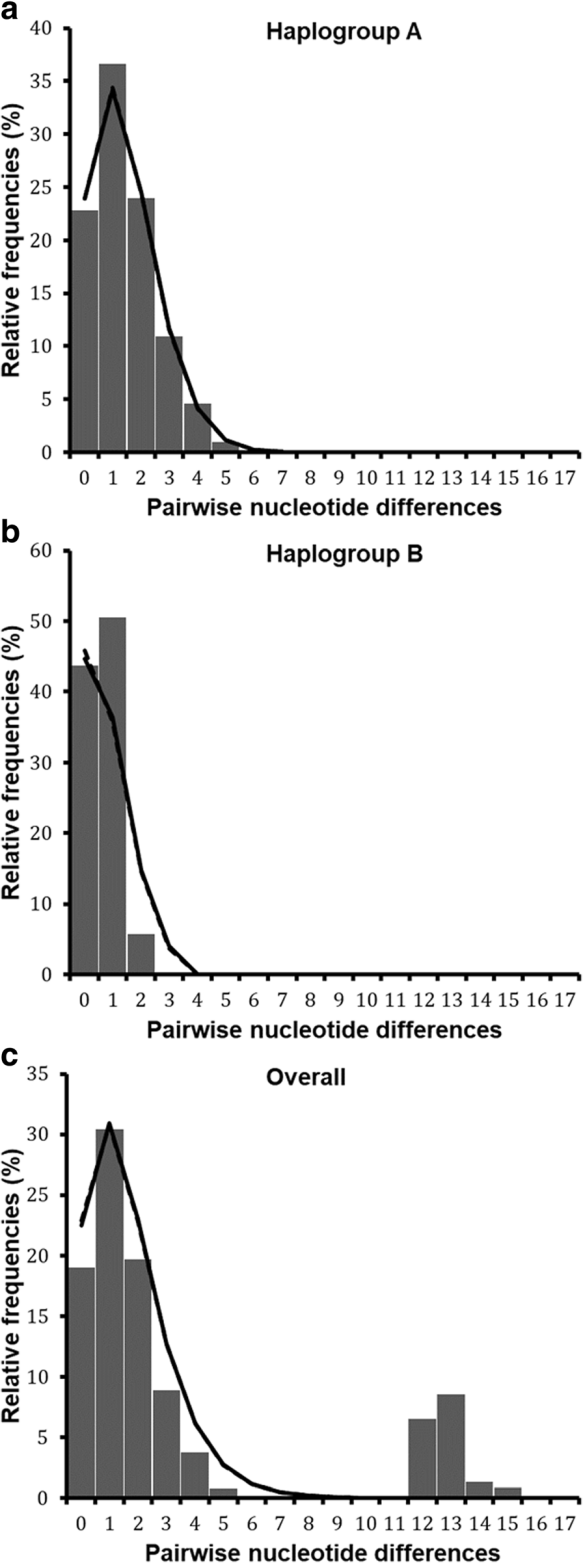
*cox*1 mismatch distribution pattern for two haplogroups of *R. appendiculatus*. **a** shows the overall mismatch distribution pattern for all AEZs, **b** and **c** show the mismatch distribution of nucleotide sequences in haplogroups A and B, respectively. The x-axis shows the number of pairwise differences between pairs of haplotype sequences and the y-axis shows their frequencies (in %). The observed frequencies are represented by solid histograms and the simulated mismatch distributions expected under demographic expansion (solid black line) and under spatial expansion (dotted black line). Simulated curves under range and demographic expansion have same pattern in these figures, they overlapped

**Fig. 6:**
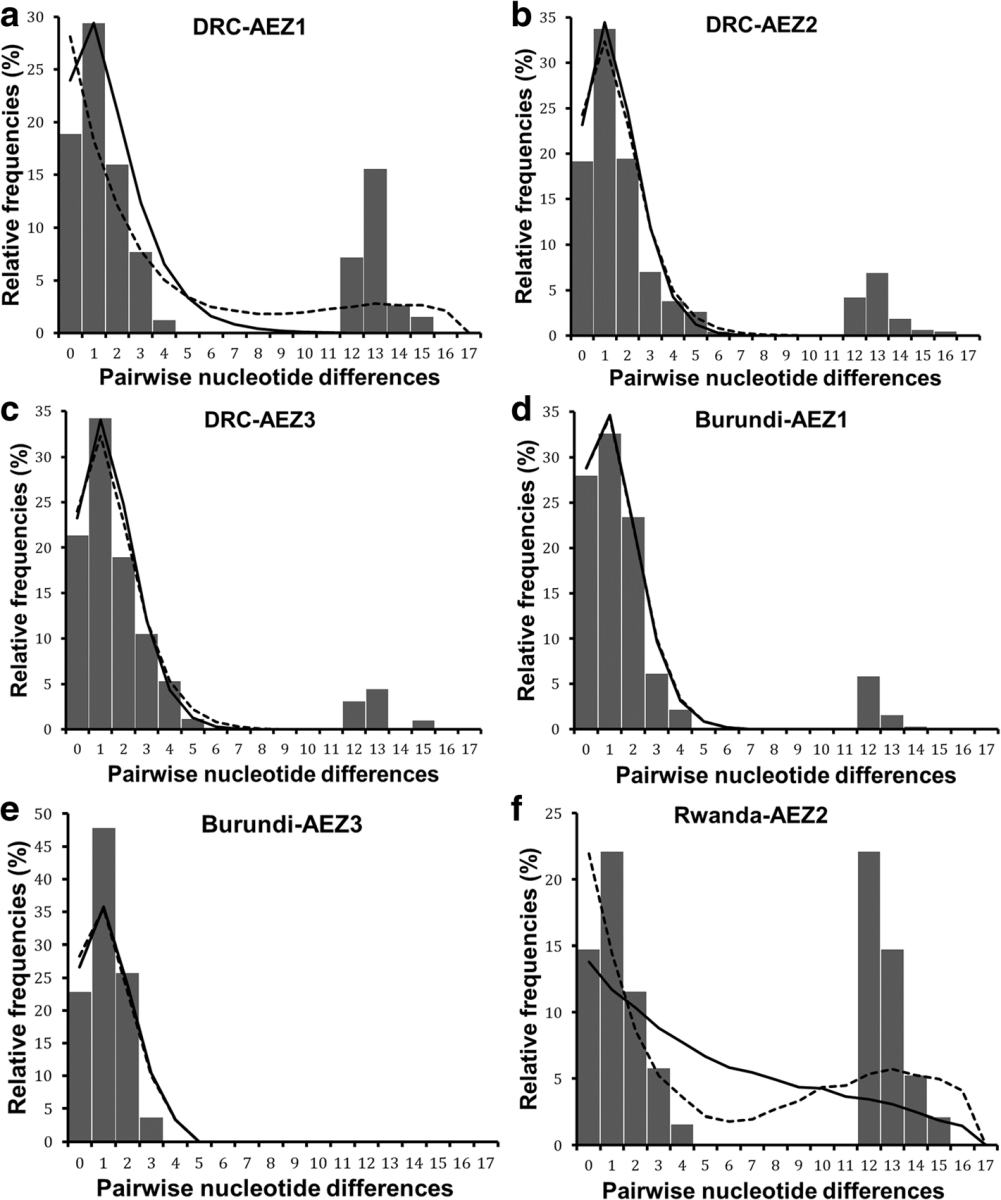
*cox*1 mismatch distribution pattern for six populations of *R. appendiculatus*. **a**, **b** and **c** show the mismatch distribution pattern for *R. appendiculatus* from DRC AEZs (AEZ1, 2 and 3, respectively); **d** and **e** represent the mismatch pattern of ticks from Burundi AEZ1 and AEZ3, respectively; **f** depicts the mismatch distribution of ticks from Rwanda AEZ2. The x-axis shows the number of pairwise differences between pairs of haplotype sequences and the y-axis shows their frequencies in %. The observed frequencies are represented by solid histograms. Black full line represents the expected distribution under sudden expansion model, and dotted line represents the distribution simulated under spatial expansion model. Simulated curves under spatial and demographic expansion have same pattern in (**d**), and they overlapped

Demographic and spatial dynamics inferred from pairwise nucleotide differences revealed bimodal pattern for the total dataset (Fig. 5a). Tajima’s *D* and Fu’s *Fs* were negative but not significant (Table 3). However, the sum of square deviation (*SSD*) and raggedness index (*RI*) for both demographic and range expansion did not deviate significantly from that expected under expansion model. The negative values of neutrality tests and the non-significance of *SSD* and *RI* indices, suggest a sudden expansion of *R. appendiculatus* populations in the Great Lakes region. The population dynamics history was also analysed separately for each of the six AEZs (Fig. 6, Additional file 5: Table S5). Most AEZs showed a bimodal mismatch distribution, except in Burundi AEZ3 where the mismatch pattern was unimodal (Fig. 6e). The observed bimodal pattern suggested population subdivision as shown by the existence of two well resolved haplogroups (Fig. 2).

The population dynamics were then inferred for each haplogroup separately (Table 3). A unimodal distribution was observed in both haplogroups (Fig. 5b, c). For the haplogroup A, we detected significant evidence of demographic and spatial expansion events from the unimodal mismatch distribution, together with significantly negative values for Tajima’s *D* (*D* = -1.5, *P* = 0.032) and Fu’s *Fs* (*Fs* = -10.4, *P* = 0.001) statistics. A good fit of sudden population expansion was also observed in this haplogroup, based on sum of squared deviation values that were not significant in all the cases: demographic (*SSD* = 0.0008, *P* = 0.44) and range (*SSD* = 0.0008, *P* = 0.26) expansion, with no significant variation of the raggedness index for both models (Table 3). In contrast, haplogroup B did not display any significant signature of expansion from the selective neutrality tests. In addition, the observed mismatch pattern for this haplogroup deviated significantly from that expected under population expansion scenario (*SSD* = 0.029, *P* = 0.049 and *P* = 0.011 for demographic and spatial expansion, respectively), implying that haplogroup B did not experience any historical population expansion. This group is characterised either by a constant population size (demographic equilibrium) or had experienced a weak signal of population bottleneck that reduced its diversity. When analysing the demographic dynamics for samples belonging to haplogroup A in each AEZ (Table 3), population expansion signal was confirmed in all AEZs by mismatch analyses exhibiting unimodal distribution (Additional file 6: Figure S1). For the six AEZs, none of the statistical comparisons between the observed and the expected distributions rejected the sudden and range expansion assumptions based on the raggedness index and the sum of squared deviation. The neutrality indices were generally negative but not significant, except in Burundi AEZ1 where both Tajima’s *D* (*D* = -1.4, *P* = 0.049) and Fu’s *Fs* (*Fs* = -2.6, *P* = 0.027) statistics showed significant negative values and in Rwanda AEZ2 where Fu’s *Fs* was negative (*Fs* = -2.1) and significant (*P* = 0.041). According to the mismatch distribution and negative values for neutrality tests, the hypothesis of population growth and spatial expansion models could not be rejected in the six AEZs, which was consistent with a model of sudden expansion for each population subdivision.

### Phylogeographical structure

To study the phylogeographical structure of *R. appendiculatus* in Africa, the haplotype sequences found in this study along with those retrieved from GenBank (Additional file 1: Table S1) were used to reconstruct the phylogenetic using ML tree and the MJ network methods based on *cox*1 and *12S* rRNA genes. Thirty-six *cox*1 haplotypes were identified in 105 sequences including 50 haplotypes from GenBank and the 55 haplotype sequences obtained in our study for each of the six AEZs and for Tanzania (Table 5). Twenty-eight haplotypes had been already described in different African countries, and eight haplotypes (CH3, CH9, CH10, CH14, CH16, CH18, CH20 and CH33) were newly described in the present study. Most of these new haplotypes were less abundant (Additional file 3: Table S3) and diverged from the common ancestral haplotypes generally by only one substitution (Fig. 4). Haplotypes CH1, CH7, CH11 were the most ubiquitous and shared wide geographical distribution in affected African countries. CH1 haplotype was shared by Kenya, Eastern Zambia, DRC (AEZ1, 2 and 3), Burundi (AEZ1 and 3), Tanzania and Rwanda (AEZ2 and GenBank sequences), while haplotype CH7 was reported in Kenya, South Africa, Zimbabwe, Grande Comore, Eastern and southern provinces of Zambia, DRC (AEZ1, 2 and 3), Burundi (AEZ1) and Rwanda (AEZ2). Haplotype CH11 was present in Kenya, Rwanda (AEZ2 and GenBank sequences), Comoros and DRC (AEZ1, 2 and 3). Eighteen haplotypes mostly with unique sequences were found to be restricted to Kenya and have not been reported in any other country. In the same way, haplotype CH23 was particular to Uganda. This country did not share any haplotype with other countries. The ML phylogenetic tree reconstructed using the 105 sequences indicated that globally, the African tick *R. appendiculatus* is consistently clustered into two groups (haplogroups A and B) well-supported by a ML bootstrap value of 100% (Fig. 3). Our 19 haplotypes that had been described for haplogroup A (Table 2, Additional file 3: Table S3) formed one clade with 19 haplotypes from Kenya (19/29), all the 3 haplotypes from Rwanda (3/3), six haplotypes from Eastern province of Zambia (6/7), all the five haplotypes from Tanzania, whereas our three haplotypes of haplogroup B were clustered with 10 haplotypes from Kenya, the single haplotype from Grande Comore, all haplotypes from South Africa (3/3), Zimbabwe (3/3), Uganda (2/2), Southern province of Zambia (2/2), and one haplotype from Eastern province of Zambia. In addition to ML tree, the MJ network also revealed that *R. appendiculatus* is divided into two main groups in Africa, separated by seven mutational steps (Fig. 4).

**Table 5:** *Rhipicephalus appendiculatus cox*1 haplotypes and their distribution among agro-ecological zones of the Great lakes region and other sub-Saharan African countries

Haplotype^d^	Haplotypes from GenBank: Country (original haplotype name and GenBank number)	Present study	Haplogroup
CH1^a^	Kenya (H2: KU725891, H4: KU725893, H6: KU725895)^e^, Rwanda (H3:DQ901360)^f^ Zambia-east (H4: KX276942^e^, H5: DQ859266^g^, H3:DQ901361^f^, H2: DQ859265^g^)	Burundi (AEZ1, AEZ3), DRC (AEZ1, AEZ2, AEZ3), Tanzania (TZ18, TZ10, TZ08, TZ20), Rwanda (AEZ2)	A
CH3	–	Burundi AEZ1	A
CH4	Rwanda (H6: DQ901362)^f^	Burundi AEZ1, Rwanda (AEZ2)	A
CH6^b^	Kenya (H5: KU725894)^e^	Burundi (AEZ1, AEZ3), DRC (AEZ1, AEZ2)	A
CH7^c^	Kenya (H1: KU725890)^e^, South Africa (H1: KX276939^e^, H1: KX276940^e^, H1: DQ901356^f^), Zambia-east (H1: DQ859261)^g^, Zambia-south (H1:KX276943^e^, H1: DQ859262)^g^, Zimbabwe (AF132833^h^, KC503257^i^, H1: KX276944^e^), Grande Comore (H1)^j^	Burundi AEZ1, DRC (AEZ1, AEZ2, AEZ3), Rwanda (AEZ2)	B
CH9	–	Burundi AEZ3	A
CH10	–	Burundi AEZ1	A
CH11^d^	Kenya (H3: KU725892)^e^, Rwanda (H3: DQ901363)^f^, Grande Comore (H3)^j^	DRC (AEZ1, AEZ2, AEZ3), Rwanda (AEZ2)	A
CH12	Kenya (H11: KU725900)^e^	DRC (AEZ2, AEZ3)	A
CH14	–	DRC AEZ3	A
CH16	–	DRC AEZ1	A
CH18	–	DRC AEZ2	A
CH20	–	DRC AEZ2	B
CH23	Uganda (H8: KX276941, KU725897)^e^	–	B
CH24	Kenya (H14: KU725903)^e^	–	B
CH25	Kenya (H27: KU725916)^e^	–	B
CH26	Grande Comore (H2: DQ901357)^f,j^, Kenya (H7: KU725896, H13: KU725902)^e^	–	B
CH27	Kenya (H16: KU725905)^e^	–	B
CH28	Kenya (H21: KU725910^e^, H9: DQ901359^f^, H9: DQ901358^f^)	–	B
CH29	Kenya (H28: KU725917)^e^	–	B
CH30	Kenya (H9: KU725898)^e^	–	A
CH31	Kenya (H17: KU725906^e^	–	A
CH32	Kenya (H24: KU725913)^e^	–	A
CH33	–	Tanzania (TZ13)	A
CH34	Kenya (H22: KU725911)^e^	–	A
CH35	Kenya (H23: KU725912)^e^	–	A
CH36	Kenya (H10: KU725899)^e^	–	A
CH37	Kenya (H15:KU725904)^e^	–	A
CH38	Kenya (H20: KU725909)^e^	–	A
CH39	Kenya (H19: KU725908)^e^	–	A
CH40	Kenya (H26: KU725915)^e^	–	A
CH41	Kenya (H25: KU725914^e^	–	A
CH42	Kenya (H18: KU725907)^e^	–	A
CH43	Kenya (H12: KU725901)^e^	–	A
CH44	Zambia-east (H3: DQ859263)^g^	–	A
CH45	Zambia-east (H4: DQ859264)^g^	–	A

^a^CH1: variants CH1, CH2, CH5, CH15, CH17 and CH21 (Additional file 3: Table S3)

^b^CH6: variants CH6, CH8 and CH22 (Additional file 3: Table S3)

^c^CH7: variants CH7 and CH13; d CH11: CH11 and CH19 (Additional file 3: Table S3)

^d^Haplotypes underlined are exclusive to the Great Lakes region. Similar data for *12S* rRNA are detailed in Additional file 7: Table S6

^e^[31]; ^f^ [29]; ^g^ [52]; ^h^[68]; ^i^[69]; ^j^[14]

Similar findings were confirmed by *12S* rRNA gene. Our 23 *12S* rRNA individual haplotypes from each of the six AEZs were analysed together with the 29 sequences obtained from GenBank (Additional file 1: Table S1). Fourteen haplotypes were observed, two most common (12SH1 and 12SH5) and 12 minors (defined mostly by one sequence or restricted to particular country) (Additional file 7: Table S6). Haplotype 12SH1 was common in DRC, Burundi, Rwanda, Kenya and Eastern province of Zambia, while haplotype 12SH5 was present in DRC, Rwanda, Zimbabwe, Comoros, South Africa, Eastern Zambia and Kenya. Six new haplotypes were not found outside the Great lakes region (12SH3, 12SH4 and 12SH6-H9). The NJ phylogenetic resolved these *12S* rRNA haplotypes into two haplogroups (haplogroup A and B) supported by 97% bootstrap value (Additional file 8: Figure S2). Their pattern was identical to that observed from *cox*1 haplogroups. However, the Ugandan haplotype (12SH10: GenBank AF150028) was clustered in haplogroup A, showing that the haplogroup A is also present in Uganda. Unfortunately, we did not find its corresponding *cox*1 sequence.

## Discussion

This study analysed the intraspecific variation of mitochondrial DNA to better understand how factors such as agro-ecological zones and anthropogenic movements of cattle may affect population genetic structure and population expansion history of the tick *R. appendiculatus*, the main vector of *T. parva* in sub-Saharan Africa. We expected evidence of *R. appendiculatus* population expansion, gene flow and different colonization patterns of tick lineages, facilitated by the reported cattle mobility in the Great Lakes region.

### Two *R. appendiculatus* lineages that are more variable in lowlands than highlands occur in the Great Lakes region

The 22 haplotypes identified by DNA polymorphic analysis of *cox*1 gene locus were clustered into two well-defined major groups, named haplogroup A (the most frequent) and haplogroup B. Similar grouping were obtained with *12S* rRNA analyses. The two haplogroups identified in the present study have been previously described as “east African” and “southern African” genetic groups [29], corresponding to our haplogroups A and B, respectively. This genetic grouping fitted well with the phenotypical, physiological and ecological variation studies which have previously distinguished two major subpopulations of *R. appendiculatus* in Africa [32–37, 51]. These phenotypic and physiological variations are largely associated to agro-ecological and geographical subdivisions. Tropical areas with prolonged and marked dry seasons are more suitable for larger sized ticks expressing high intensity of diapause and displaying univoltine phenology, corresponding to “southern African group” or haplogroup B. Equatorial areas with bimodal or continuous rainfall rather harbour smaller ticks without diapause with bivoltine or continuous phenology, corresponding to “east African group” or haplogroup A [37, 52].

The highest genetic variability was observed in lowlands, whereas a relatively lower diversity was observed in midlands and highlands. The high genetic diversity in lowlands can be explained by the strong presence of the two lineages A and B observed in these AEZs. The coexistence of these lineages could originate from the dispersal of the tick through livestock movement between AEZs [53], associated to the suitability of semi-arid climate for lineage B expressing obligatory diapause [37].

### Moderate genetic structure of *R. appendiculatus* between lowlands and highlands

Population genetic analyses of *cox*1 gene variation in *R. appendiculatus* revealed low to moderate genetic differentiation values and high gene flow rates among AEZs. The two identified *R. appendiculatus* lineages were sympatric in the Great Lakes region, although lineage A was the most abundant and widely distributed in all AEZs and lineage B was particular confined to lowlands, where the climate is tropical dry, more arid with lower annual rainfall and longer dry season than in highlands (short dry season with abundant annual rainfall). These climate conditions in lowlands are quite similar to the described ecological zones of lineage B in southern Africa [29, 52]. In addition, altitudinal gradient seems to be a key factor that shapes the distribution pattern and the establishment ability of lineage B. Its frequency decreases with increasing altitude. High degree of genetic similarity was observed between the lowlands of DRC and the low plateau of Rwanda and between the highlands of DRC and Burundi, which are geographically distant from each other. The most likely explanation for this is that the spatial pattern of *R. appendiculatus* lineages is not only driven by geographical separation as described in previous studies [52], but also related to their ecological preferences, as observed by the significant genetic differentiation among lowlands and highlands AEZs. On the other hand, adjacent AEZs shared more migrants, especially of lineage A, facilitated by short-distance seasonal movement of cattle [9], which may have reduced the geographical structuring of the tick [11, 18]. Analysis of molecular variance (AMOVA) confirmed these findings showing that the variance explained by divergence between the six AEZs was lower (6%), while the largest fraction of genetic variation was observed among individuals within AEZs (94%).

### *Rhipicephalus appendiculatus* lineage A has undergone sudden demographic and range expansion in the Great Lakes region

The demographic and spatial dynamics were analysed using multiple algorithms, to elucidate colonization events of the tick *R. appendiculatus* that took place in the Great Lakes region. A strong evidence of recent spatial and demographic expansion was observed for lineage A in all AEZs included in the study. Analyses of *cox*1 sequences revealed relatively high haplotype diversity contrasted with low nucleotide diversity values for each population, suggesting a sudden population expansion [54, 55]. This result, together with negative values of Tajima’s *D* and Fu’s *Fs*, the star-like radiation, the unimodal mismatch pattern and non-significant *RI* and *SSD* statistics, further support the recent evolutionary history and sudden population growth experienced by lineage A [56–58]. We did not estimate the expansion time, but we hypothesize that the expansion was recent and not sufficient to increase the nucleotide diversity, probably because of recent coalescence, while rapid population expansion following a selective sweep (bottleneck or genetic drift) could have accumulated new mutations that sufficiently increased the haplotype diversity [45, 59, 60]. This could explain the excess of singletons polymorphic sites and rare haplotypes that diverge from ancestral haplotypes by only 1–2 mutational steps [45, 61]. However, the strong bimodal mismatch pattern observed in Rwanda AEZ2 and DRC AEZ1 suggests the coexistence of *R. appendiculatus* lineages after recent colonization events or exchanging migrants [45, 56, 62]. The scenario of lineage B contrasts with that of lineage A. Analyses indicate, for lineage B, an equilibrium situation that is characterised by a weak signal of recent bottleneck and no evidence of population expansion. It was also less diverse than haplogroup A, indicating that haplogroup A is experiencing population expansion independently of haplogroup B and it has been established longer in the Great Lakes region, while haplogroup B was probably introduced more recently and established a founder population. There are three possible explanations of the equilibrium observed in haplogroup B: (i) only few haplotypes were recently introduced; (ii) only the few identified haplotypes persisted after a bottleneck; or (iii) haplogroup B is experiencing an initial selection sweep which has reduced the number of rare haplotypes and singleton mutations [56]. When we analysed *R. appendiculatus cox*1 sequences available in Africa, the star radiation of the MJ network in each group suggests that the two lineages went through a demographic expansion and evolve independently of each other with limited gene exchange. Unfortunately, we were not able to test the hypothesis of crossbreeding events between the two lineages described here, because of the maternal inheritance of mitochondrial genes used in the present study. More studies, such as extensive biological characterisation, crossbreeding experiments and the use of biparental inheritance markers are necessary to investigate the panmixia of the two lineages and the genetic mechanisms driving their establishment and corresponding phenotypic variations in changed environments.

The fact that *R. appendiculatus* has undergone spatial expansion was in accordance with the expectation that long and short distance movement of cattle are key factors of spreading ticks. The processes responsible for that evolutionary pattern may have resulted, not only from range expanding of the previously established haplotypes to proximate AEZs, but also from the recolonization events of ticks from other regions and countries [53, 63]. In addition to cattle movement, the population expansion and establishment of novel haplotypes in previously unoccupied areas could have been driven by recent environmental and climatic changes, affecting vector-borne diseases landscape over recent decades [64–66].

### Sympatric and allopatric ecological zones of *R. appendiculatus* lineages in Africa

Phylogenetic trees produced two main genetic subpopulations of *R. appendiculatus* that have a wide distribution range in Africa, with large divergence in behavioural diapause [36, 37], spatial variation in body size [35, 51], ecology and phenology [32–34]. Initially, the two lineages were considered as “east African” and “southern African” genetic groups [29]. To date, they are sympatric in most eastern and central African ecological zones. For instance, previous studies did not reveal the presence of haplogroup B in Rwanda [29]. This could be an indication of recent introduction of the tick in the Great Lakes region. The MJ network further elucidate that the initial population of haplogroup B could have come from an ancestral female of haplotype CH7, which is the most prevalent haplotype of this group in areas where it occurs. Consequently, two different eco-genetic zones are shaped in Africa, the sympatric zone where the two lineages are found, which covers central and eastern Africa, and allopatric zone in southern Africa where the two lineages have clear geographical and ecological separation. For instance, in Zambia, lineage B is present in the south (long dry season) and lineage A in the east of the country (shorter dry season) [29]. In Grande Comore, lineage B has established a stable population, while lineage A was found on imported cattle [14]. In areas where the two lineages are sympatric, their respective abundances differ, mainly driven by their divergence in ecological preferences and plasticity [33]. The sympatric relationship is in agreement with the observation made by Berkvens et al. [67], where an east African stock from Kenya expressed diapause, contrasting with the result obtained by Madder et al. [36], where another stock from the same region was unable to enter diapause. These evidences show that lineage B has a greater invasive ability into new habitats and better fit wide range of tropical and equatorial conditions, while lineage A is particularly confined to equatorial conditions. This could be explained by the larger body size and obligatory diapause behaviour for southern African ticks, which allow them to survive hot and dry ecological conditions [33, 37]. We hypothesise that these characteristics make the development of lineage B slower than lineage A in sympatric areas, giving an evolutionary advantage to the latter. This could also reduce the abundance of lineage B delaying its oviposition during unfavourable conditions [37]. The processes that took place to divide the two groups need to be further investigated. However, we propose that they could have diverged following genetic drift due to founder events of natural geographical barrier that may result in reproductive isolation. It is demonstrated that biogeographical break again host migration reduces gene exchange and could dictate the spatial and reproductive separation within a species [60].

## Conclusions

This study provided new insights into the genetic structure and colonization events of *R. appendiculatus* in the Great Lakes region and over its distribution range in sub-Saharan Africa. Our results highlighted the occurrence of two major genetic lineages (A and B) in the Great Lakes region. The two lineages are not spatially structured in the study region and they differ in their colonization histories and pattern. Lineage B, not previously reported, was probably introduced recently in the region and its occurrence decreases with increased altitude, whereas lineage A, widely distributed, has been longer established and subjected to sudden demographic and spatial expansion most likely related to short and long-distance cattle movement. *Rhipicephalus appendiculatus* ticks are more diverse in lowlands than highlands with moderate genetic differentiation between the two ecosystems, while more genetic similitude is found in zones with same agro-ecological profiles, in spite of their geographical distance. The genetic distribution of *R. appendiculatus* suggests two different eco-genetic zones in Africa, the sympatric zone (central and eastern Africa) where the two lineages coexist and the allopatric zones (southern Africa) where they have clear geographical divergence. The range expansion pattern of lineages and the genetic admixture of *R. appendiculatus* populations observed in the Great Lakes region can strongly affect the epidemiological dynamics of ECF. This could partially explain the endemic instability and occasional epidemics due to the introduction and temporal subsistence of infected ticks mostly in fringes areas of lowlands.

## Additional files

Additional file 1: **Table S1.** *Rhipicephalus appendiculatus cox*1 and *12S* rRNA haplotype sequences retrieved from GenBank. (DOCX 16 kb)

Additional file 2: **Table S2.** *cox*1 and *12S* rRNA BLAST results for species identification and confirmation. (DOCX 16 kb)

Additional file 3: **Table S3.** Polymorphism in the 22 haplotypes of the *cox*1 gene fragment of *R. appendiculatus*. (DOCX 21 kb)

Additional file 4: **Table S4.** Population genetic structure inferred by analysis of molecular variance (AMOVA) based on *cox*1 sequences of *R. appendiculatus* from different agro-ecological zones. (DOCX 13 kb)

Additional file 5: **Table S5.** Evolutionary neutrality, demographic and spatial history of mitochondrial *cox*1 gene. (DOCX 15 kb)

Additional file 6: **Figure S1.** *cox*1 mismatch distribution pattern for *R. appendiculatus* haplogroup A in different agro-ecological zones. (DOCX 193 kb)

Additional file 7: **Table S6.*** Rhipicephalus appendiculatus 12S* rRNA haplotypes and their distribution among agro-ecological zones of the Great lakes region and other sub-Saharan African countries. (DOCX 15 kb)

Additional file 8: **Figure S2.** Neighbor-joining tree of *12S* haplotype sequences for *R. appendiculatus* across African countries. (DOCX 18 kb)

## Abbreviations

AMOVA: Analysis of molecular variance
BecA: Biosciences eastern and central Africa
BLAST: Basic local alignment search tool
*cox*1: Cytochrome *c* oxidase subunit 1
*D*: Tajima’s neutrality test
ECF: East Coast fever
*Fs*: Fu’s neutrality tests
*F*_*ST*_: Right’s fixation index
*h*: Number of haplotypes
*Hd*: Haplotype diversity
ILRI: International Livestock Research Institute
ITS2: Internal transcribed spacer 2
*K*: Mean number of pairwise nucleotide differences within population
*K*_*XY*_: Average number of nucleotide differences between populations
ML: Maximum likelihood
NCBI: National Center for Biotechnology Information
NJ: Neighbor-joining
*Nm*: Number of migrants
PCR: Polymerase chain reaction
PIS: Parsimony informative sites
*RI*: Harpending’s raggedness index
rRNA: ribosomal RNA
*S*: Segregating sites
SD: Standard deviation
SSD: Sum of squares deviation
π: nucleotide diversity
