# Supplementary material for: Mitochondrial phylogeography and population structure of the cattle tick Rhipicephalus appendiculatus in the African Great Lakes region
Source: Parasit Vectors. 2018 May 31;11:329. doi: 10.1186/s13071-018-2904-7 (PMC5984310; doi:10.1186/s13071-018-2904-7)
Supplement: Supplementary file 1 — Table S1. Rhipicephalus appendiculatus cox1 and 12S rRNA haplotype sequences retrieved from GenBank. (DOCX 16 kb) [file 13071_2018_2904_MOESM1_ESM.docx]

**Additional file 1: Table S1.** *Rhipicephalus appendiculatus cox1* and *12S* rRNA haplotype sequences retrieved from GenBank

| **Gene locus** | **Geographical origin** | **Number of sequences** | **GenBank**  **accession number** | **Reference** |
| --- | --- | --- | --- | --- |
| *Cox1* | Kenya | 29 | KU725890-917 | [31] |
|  |  |  | DQ901358-59 | [29] |
|  | Zimbabwe | 3 | AF132833 | [68] |
|  |  |  | KX276944 | [31] |
|  |  |  | KC503257 | [69] |
|  | Comoros | 1 | DQ901357 | [29] |
|  | Rwanda | 3 | DQ901360, DQ901362-63 | [29] |
|  | Zambia (Southern province) | 2 | DQ859262 | [52] |
|  |  |  | KX276943 | [31] |
|  | Zambia (Eastern province) | 7 | DQ859261, DQ859263-66 | [52] |
|  |  |  | DQ901361 | [29] |
|  |  |  | KX276942 | [31] |
|  | South Africa | 3 | DQ901356 | [29] |
|  |  |  | KX276939-40 | [31] |
|  | Uganda | 2 | KX276941, KU725897 | [31] |
| *12S* rRNA | Kenya | 6 | KX276945-49 | [31] |
|  |  |  | DQ901320 | [29] |
|  | Zimbabwe | 2 | AF031859, AF150027 | [73] |
|  | Comoros | 1 | DQ901317 | [29] |
|  | Rwanda | 5 | DQ901279, DQ901281-82, DQ901284, DQ901286 | [29] |
|  | Zambia (Southern province) | 6 | DQ849203-05, DQ849208, DQ901309, DQ901311 | [29] |
|  | Zambia (Eastern province) | 6 | DQ849207, DQ849210, DQ849212, DQ849214, DQ901277, DQ901288 | [29] |
|  | South Africa | 2 | DQ849233, DQ849235 | [29] |
|  | Uganda | 1 | AF150028 | [70] |

[29].  Mtambo J, Madder M, Van Bortel W, Geysen D, Berkvens D, Backeljau T. Genetic variation in *Rhipicephalus appendiculatus* (Acari: Ixodidae) from Zambia: correlating genetic and ecological variation with *Rhipicephalus appendiculatus* from eastern and southern Africa. J Vector Ecol. 2007;32:168-75.

[31]. Kanduma EG, Mwacharo JM, Githaka NW, Kinyanjui PW, Njuguna JN, Kamau LM, et al. Analyses of mitochondrial genes reveal two sympatric but genetically divergent lineages of *Rhipicephalus appendiculatus* in Kenya. Parasit Vectors. 2016;9:353.

[52]. Mtambo J, Madder M, Van Bortel W, Chaka G, Berkvens D, Backeljau T. Further evidence for geographic differentiation in *R. appendiculatus* (Acari: Ixodidae) from Eastern and Southern provinces of Zambia. Exp Appl Acarol. 2007;41:129-38.

[68]. Murrell A, Campbell NJH, Barker SC. Phylogenetic Analyses of the Rhipicephaline Ticks Indicate That the Genus Rhipicephalus Is Paraphyletic. Mol Phylogenet Evol. 2000;16:1-7.

[69]. Burger TD, Shao R, Barker SC. Phylogenetic analysis of mitochondrial genome sequences indicates that the cattle tick, *Rhipicephalus (Boophilus) microplus*, contains a cryptic species. Mol Phylogenet Evol. 2014;76:241-53.

[70] Beati L, Keirans JE. Analysis of the systematic relationships among ticks of the genera *Rhipicephalus and Boophilus* (Acari: Ixodidae) based on mitochondrial 12S ribosomal DNA gene sequences and morphological characters. J Parasitol. 2001;87:32-48.
