# Supplementary material for: Mitochondrial phylogeography and population structure of the cattle tick Rhipicephalus appendiculatus in the African Great Lakes region
Source: Parasit Vectors. 2018 May 31;11:329. doi: 10.1186/s13071-018-2904-7 (PMC5984310; doi:10.1186/s13071-018-2904-7)
Supplement: Supplementary file 2 — Table S2. cox1 and 12S rRNA BLAST results for species identification and confirmation. (DOCX 16 kb) [file 13071_2018_2904_MOESM2_ESM.docx]

**Additional file 2: Table S2** *cox1* and *12S* rRNA BLAST results for species identification and confirmation

| **Gene locus** | **Haplotype** | **GenBank accession no.** | **BLAST results** | | | **Reference** |
| --- | --- | --- | --- | --- | --- | --- |
|  |  |  | **GenBank match** | **Identity**  **(%)** | **Source** |  |
| *cox1* | CH1 | MF458950 | KU725895 | 100 | Kenya | [31] |
|  | CH2 | MF458951 | KU725893 | 100 | Kenya | [31] |
|  | CH3 | MF458952 | KU725895 | 99 | Kenya | [31] |
|  | CH4 | MF458953 | KU725895 | 99 | Kenya | [31] |
|  | CH5 | MF458954 | KU725891 | 100 | Kenya | [31] |
|  | CH6 | MF458955 | KU725894 | 99 | Kenya | [31] |
|  | CH7 | MF458956 | KC503257 | 100 | Zimbabwe | [69] |
|  | CH8 | MF458957 | KU725894 | 100 | Kenya | [31] |
|  | CH9 | MF458958 | KU725893 | 99 | Kenya | [31] |
|  | CH10 | MF458959 | KU725895 | 99 | Kenya | [31] |
|  | CH11 | MF458960 | KU725892 | 100 | Kenya | [31] |
|  | CH12 | MF458961 | KU725900 | 99 | Kenya | [31] |
|  | CH13 | MF458962 | AF132833 | 100 | Zimbabwe | [68] |
|  | CH14 | MF458963 | KU725894 | 99 | Kenya | [31] |
|  | CH15 | MF458964 | KU725895 | 99 | Kenya | [31] |
|  | CH16 | MF458965 | KU725895 | 99 | Kenya | [31] |
|  | CH17 | MF458966 | KU725895 | 99 | Kenya | [31] |
|  | CH18 | MF458967 | KU725893 | 99 | Kenya | [31] |
|  | CH19 | MF458968 | KU725892 | 99 | Kenya | [31] |
|  | CH20 | MF458969 | KC503257 | 99 | Zimbabwe | [70] |
|  | CH21 | MF458970 | KU725891 | 99 | Kenya | [31] |
|  | CH22 | MF458971 | KU725894 | 99 | Kenya | [31] |
| *12S* rRNA | 12SH1 | MF479189 | DQ849209 | 100 | Zambia(east) | [29] |
|  | 12SH2 | MF479190 | AF150028 | 100 | Uganda | [70] |
|  | 12SH3 | MF479191 | DQ849209 | 99 | Zambia(east) | [29] |
|  | 12SH4 | MF479192 | DQ849203 | 99 | Zambia(south) | [29] |
|  | 12SH5 | MF479193 | DQ849203 | 99 | Zambia(south) | [29] |
|  | 12SH6 | MF479194 | DQ849209 | 99 | Zambia(east) | [29] |
|  | 12SH7 | MF479195 | DQ849209 | 99 | Zambia(east) | [29] |
|  | 12SH8 | MF479196 | DQ849209 | 99 | Zambia(east) | [29] |
|  | 12SH9 | MF479197 | DQ849209 | 99 | Zambia(east) | [29] |

[29].  Mtambo J, Madder M, Van Bortel W, Geysen D, Berkvens D, Backeljau T. Genetic variation in *Rhipicephalus appendiculatus* (Acari: Ixodidae) from Zambia: correlating genetic and ecological variation with *Rhipicephalus appendiculatus* from eastern and southern Africa. J Vector Ecol. 2007;32:168-75.

[31]. Kanduma EG, Mwacharo JM, Githaka NW, Kinyanjui PW, Njuguna JN, Kamau LM, et al. Analyses of mitochondrial genes reveal two sympatric but genetically divergent lineages of *Rhipicephalus appendiculatus* in Kenya. Parasit Vectors. 2016;9:353.

[69]. Burger TD, Shao R, Barker SC. Phylogenetic analysis of mitochondrial genome sequences indicates that the cattle tick, *Rhipicephalus (Boophilus) microplus*, contains a cryptic species. Mol Phylogenet Evol. 2014;76:241-53.

[68]. Murrell A, Campbell NJH, Barker SC. Phylogenetic Analyses of the Rhipicephaline Ticks Indicate That the Genus Rhipicephalus Is Paraphyletic. Mol Phylogenet Evol. 2000;16:1-7.

[70] Beati L, Keirans JE. Analysis of the systematic relationships among ticks of the genera Rhipicephalus and Boophilus (Acari: Ixodidae) based on mitochondrial 12S ribosomal DNA gene sequences and morphological characters. J Parasitol. 2001;87:32-48.
