# Supplementary material for: Mitochondrial phylogeography and population structure of the cattle tick Rhipicephalus appendiculatus in the African Great Lakes region
Source: Parasit Vectors. 2018 May 31;11:329. doi: 10.1186/s13071-018-2904-7 (PMC5984310; doi:10.1186/s13071-018-2904-7)
Supplement: Supplementary file 3 — Table S3. Polymorphism in the 22 haplotypes of the cox1 gene fragment of R. appendiculatus. (DOCX 21 kb) [file 13071_2018_2904_MOESM3_ESM.docx]

**Additional file 3: Table S3.** Polymorphism in the 22 haplotypes of the *cox1* gene fragment of *R. appendiculatus*

| **Haplotype** | **No. of sequences (%)^a^** | **Polymorphic sites (27 positions) *** | | | | | | | | | | | | | | | | | | | | | | | | | | | | **Haplogroup** | |
| --- | --- | --- | --- | --- | --- | --- | --- | --- | --- | --- | --- | --- | --- | --- | --- | --- | --- | --- | --- | --- | --- | --- | --- | --- | --- | --- | --- | --- | --- | --- | --- |
|  |  | **21** | **41** | **56** | **80** | **93** | **95** | **104** | **107** | **143** | **164** | **167** | **179** | **233** | **249** | **302** | **314** | **320** | **329** | **395** | **401** | **458** | **470** | **498** | **500** | **527** | **539** | **548** |  | |  |
| CH1 | 56 (27) | C | A | A | T | G | T | C | T | A | T | C | G | T | T | C | T | T | T | G | T | G | C | C | A | G | A | A | A | |  |
| CH2 | 59 (28) | . | G | . | . | . | . | . | . | . | . | . | . | . | . | . | . | . | . | . | . | . | . | . | . | . | . | . | A | |  |
| CH3 | 1 (0.5) | . | . | . | . | . | . | . | . | . | C | . | . | . | . | . | . | . | . | . | . | . | . | . | . | . | . | . | A | |  |
| CH4 | 2 (1) | . | . | . | . | . | . | . | . | . | . | . | A | . | . | . | . | . | . | . | . | . | . | . | . | . | . | G | A | |  |
| CH5 | 39 (19) | . | G | . | . | . | . | . | . | G | . | . | . | . | . | . | . | . | . | . | . | . | . | . | . | . | . | . | A | |  |
| CH6 | 2 (1) | . | . | . | . | . | . | T | . | . | . | . | . | . | . | . | . | . | . | A | . | . | . | . | . | . | . | . | A | |  |
| CH8 | 2 (1) | . | . | . | . | . | . | . | . | . | . | . | . | . | . | . | . | . | . | A | . | . | . | . | . | . | . | . | A | |  |
| CH9 | 1 (0.5) | . | G | . | . | . | . | . | . | . | . | . | . | C | . | . | . | . | . | . | . | . | . | . | . | . | . | . | A | |  |
| CH10 | 1 (0.5) | . | . | . | . | . | . | . | . | . | . | . | . | . | . | . | . | . | . | . | . | . | . | . | . | . | G | . | A | |  |
| CH11 | 10 (5) | . | . | . | . | . | . | . | . | . | . | . | . | . | . | . | . | C | . | A | . | . | . | . | . | . | . | . | A | |  |
| CH12 | 5 (2) | . | G | . | . | . | . | . | . | G | . | . | . | . | . | . | . | . | . | . | . | . | . | . | . | A | . | . | A | |  |
| CH14 | 1 (0.5) | . | G | . | . | . | . | . | . | . | . | . | . | . | . | . | . | . | . | A | . | . | . | T | . | . | . | . | A | |  |
| CH15 | 1 (0.5) | . | . | . | . | . | . | . | C | . | . | . | . | . | . | . | . | . | . | . | . | . | . | . | . | . | . | . | A | |  |
| CH16 | 2 (1) | . | . | . | . | . | . | . | . | . | . | . | . | . | . | . | . | . | C | . | . | . | . | . | . | . | . | . | A | |  |
| CH17 | 1 (0.5) | . | . | . | C | . | . | . | . | . | . | . | . | . | . | . | . | . | . | . | . | . | . | . | . | . | . | . | A | |  |
| CH18 | 2 (1) | . | G | . | . | . | . | . | . | . | A | . | . | . | . | . | . | . | . | . | . | . | . | . | . | . | . | . | A | |  |
| CH19 | 2 (1) | . | . | . | . | . | G | . | . | . | . | . | . | . | . | . | . | C | . | A | . | . | . | . | . | . | . | . | A | |  |
| CH21 | 1 (0.5) | . | G | G | . | . | . | . | . | G | . | . | . | . | . | . | . | . | . | . | . | . | . | . | . | . | . | . | A | |  |
| CH22 | 1 (0.5) | . | G | . | . | A | . | . | . | . | . | . | . | . | . | . | . | . | . | A | . | . | . | . | . | . | . | . | A | |  |
| CH7 | 8 (4) | T | T | . | . | . | . | T | . | . | . | T | . | . | C | T | C | . | . | . | C | A | T | . | G | T | . | . | B | |  |
| CH13 | 11 (5) | T | T | . | . | . | . | T | . | G | . | T | . | . | C | T | C | . | . | . | C | A | T | . | G | T | . | . | B | |  |
| CH20 | 1 (0.5) | T | T | . | . | . | . | T | . | . | . | T | . | C | C | T | C | . | . | . | C | A | T | . | G | T | . | . | B | |  |

*^a^* Number of specimens sharing the same haplotype

*The number represents the position of variable sites (bp)

dots (.) represent nucleotides identical to haplotype 1
