# Supplementary material for: Mitochondrial phylogeography and population structure of the cattle tick Rhipicephalus appendiculatus in the African Great Lakes region
Source: Parasit Vectors. 2018 May 31;11:329. doi: 10.1186/s13071-018-2904-7 (PMC5984310; doi:10.1186/s13071-018-2904-7)
Supplement: Supplementary file 4 — Table S4. Population genetic structure inferred by analysis of molecular variance (AMOVA) based on cox1 sequences of R. appendiculatus from different agro-ecological zones. (DOCX 13 kb) [file 13071_2018_2904_MOESM4_ESM.docx]

**Additional file 4: Table S4.** Population genetic structure inferred by analysis of molecular variance (AMOVA) based on *cox1* sequences of *R. appendiculatus* from different agro-ecological zones

| Haplogroup | Level of partitioning | Source of variation | d.f. | Percentage of variation | *P-value* |
| --- | --- | --- | --- | --- | --- |
| Overall data | AEZs | Among AEZ | 5 | 6 | < 0.001 |
|  |  | Within AEZ | 203 | 94 | - |
| Haplogroup A | AEZs | Among AEZ | 5 | 6 | < 0.001 |
|  |  | Within AEZ | 183 | 94 | - |
|  | DRC | Among AEZ | 2 | 6 | < 0.001 |
|  |  | Within AEZ | 130 | 94 |  |
|  | Burundi | Among AEZ | 1 | 6 | 0.06 |
|  |  | Within AEZ | 40 | 94 | - |
