# Supplementary material for: Mitochondrial phylogeography and population structure of the cattle tick Rhipicephalus appendiculatus in the African Great Lakes region
Source: Parasit Vectors. 2018 May 31;11:329. doi: 10.1186/s13071-018-2904-7 (PMC5984310; doi:10.1186/s13071-018-2904-7)
Supplement: Supplementary file 5 — Table S5. Evolutionary neutrality, demographic and spatial history of mitochondrial cox1 gene. (DOCX 15 kb) [file 13071_2018_2904_MOESM5_ESM.docx]

**Additional file 5: Table S5.** Evolutionary neutrality, demographic and spatial history of mitochondrial *cox1* gene

| **Statistics** | **DRC** | | |  | **Burundi** | | **Rwanda** | **Overall** |
| --- | --- | --- | --- | --- | --- | --- | --- | --- |
|  | **AEZ1** | **AEZ2** | **AEZ3** |  | **AEZ1** | **AEZ3** | **AEZ2** |  |
| Selective neutrality tests |  |  |  |  |  |  |  |  |
| Tajima's D  (P-value) | 0.41  (0.71) | -0.95 (0.17) | -1.3 (0.14) |  | -2.1  (0.005)* | -0.21 (0.44) | 0.92  (0.83) | -0.93  (0.23) |
| Fu's Fs  (P-value) | 1  (0.71) | -1.4 (0.31) | 0.84 (0.7) |  | -1.6  (0.048)* | -1.1 (0.15) | 1.6  (0.8) | -3.8  (0.15) |
| Demographic expansion |  |  |  |  |  |  |  |  |
| Harpending’s Raggedness index (RI) | 0.061 | 0.064 | 0.055 |  | 0.047 | 0.16 | 0.086 | 0.049 |
| *P* (Simulated RI≥ Observed RI) | 0.89 | 0.29 | 0.47 |  | 0.79 | 0.11 | 0.28 | 0.51 |
| Sum of Squared deviation (SSD) | 0.13 | 0.014 | 0.007 |  | 0.006 | 0.022 | 0.084 | 0.017 |
| *P* (Simulated SSD≥Observed SSD) | 0.004* | 0.047* | 0.19 |  | 0.5 | 0.16 | 0.12 | 0.1 |
| Spatial expansion |  |  |  |  |  |  |  |  |
| Harpending’s Raggedness index (RI) | 0.061 | 0.064 | 0.055 |  | 0.047 | 0.16 | 0.086 | 0.049 |
| *P* (Simulated RI≥ Observed RI) | 0.73 | 0.39 | 0.54 |  | 0.78 | 0.12 | 0.59 | 0.74 |
| Sum of Squared deviation (SSD) | 0.043 | 0.014 | 0.006 |  | 0.006 | 0.022 | 0.062 | 0.036 |
| *P* (Simulated SSD≥Observed SSD) | 0.23 | 0.044* | 0.22 |  | 0.45 | 0.082 | 0.13 | 0.28 |

*Values are statistically significant at p < 0.05; Significance was determined using 1000 coalescent simulations

*Abbreviations: D*, Tajima’s neutrality statistic; *Fs*, Fu’s neutrality statistic
