# Supplementary material for: Mitochondrial phylogeography and population structure of the cattle tick Rhipicephalus appendiculatus in the African Great Lakes region
Source: Parasit Vectors. 2018 May 31;11:329. doi: 10.1186/s13071-018-2904-7 (PMC5984310; doi:10.1186/s13071-018-2904-7)
Supplement: Supplementary file 6 — Figure S1. cox1 mismatch distribution pattern for R. appendiculatus haplogroup A in different agro-ecological zones. (DOCX 193 kb) [file 13071_2018_2904_MOESM6_ESM.docx]

**Additional file 6: Figure S1.** *cox1* mismatch distribution pattern for *R. appendiculatus* Haplogroup A in different agro-ecological zones. The x-axis shows the number of pairwise differences between pairs of haplotype sequences and the y-axis shows their frequencies. The observed frequencies are represented by solid histograms and the simulated mismatch distributions expected under demographic expansion (solid black line) and under spatial expansion (dotted black line)
