# Supplementary material for: Mitochondrial phylogeography and population structure of the cattle tick Rhipicephalus appendiculatus in the African Great Lakes region
Source: Parasit Vectors. 2018 May 31;11:329. doi: 10.1186/s13071-018-2904-7 (PMC5984310; doi:10.1186/s13071-018-2904-7)
Supplement: Supplementary file 7 — Table S6. Rhipicephalus appendiculatus 12S rRNA haplotypes and their distribution among agro-ecological zones of the Great lakes region and other sub-Saharan African countries. (DOCX 15 kb) [file 13071_2018_2904_MOESM7_ESM.docx]

**Additional file 7: Table S6***. Rhipicephalus appendiculatus* *12S* rRNA and their distribution among agro-ecological zones of the Great lakes region and other sub-Saharan African countries

| **Haplotype** | **Haplotypes from GenBank: Country (original haplotype name and GenBank number)** | **This study** | **Haplogroup** |
| --- | --- | --- | --- |
| 12SH1 | Kenya (H2: KX276946, H5: KX276949) ^1^, Rwanda (H5: DQ901279, H5: DQ901281, H5: DQ901282, H5: DQ901284)^2^, Zambia-east (H5: DQ849210, DQ901288)^2^ | Burundi (AEZ1, AEZ3), DRC AEZ1, AEZ2, AEZ3), Rwanda AEZ2 | A |
| 12SH2 | Kenya (H3: KX276947) ^1^ | Burundi (AEZ1, AEZ3), DRC AEZ1, AEZ2, AEZ3), Rwanda AEZ2 | A |
| 12SH3 | - | Burundi AEZ1 | A |
| 12SH4 | - | Burundi AEZ1, DRC AEZ1, Rwanda AEZ2 | B |
| 12SH5 | Zimbabwe (AF031859, AF150027)^3^, Grande Comore (H1: DQ901317) ^2^, Zambia-south (H1: DQ849203, H1: DQ849205, H1: DQ901311, H1: DQ849208, H1: DQ849204, H1: DQ901309) ^2^, Zambia-East (H1: DQ849207) ^2^, South Africa (H1: DQ849233, H1: DQ849235) ^2^, Kenya (DQ901320^2^, H1: KX276945^1^), | DRC (AEZ1, AEZ3), Rwanda AEZ2 | B |
| 12SH6 | - | DRC AEZ3 | A |
| 12SH7 | - | DRC AEZ3 | A |
| 12SH8 | - | DRC AEZ3 | A |
| 12SH9 | - | DRC AEZ3 | A |
| 12SH10 | Uganda (AF150028)^4^ | - | A |
| 12SH11 | Zambia-east (H2: DQ901277, H2: DQ849214) ^2^ | - | A |
| 12SH12 | Zambia-east (H3: DQ849212) ^2^ | - | A |
| 12SH13 | Rwanda (H4: DQ901286) ^2^ | - | A |
| 12SH14 | Kenya (H4: KX276948) ^1^ | - | B |

^1^[31]; ^2^[29]; ^3^[73], ^4^[74]

[29]. Mtambo J, Madder M, Van Bortel W, Geysen D, Berkvens D, Backeljau T. Genetic variation in *Rhipicephalus appendiculatus* (Acari: Ixodidae) from Zambia: correlating genetic and ecological variation with *Rhipicephalus appendiculatus* from eastern and southern Africa. J Vector Ecol. 2007;32:168-75.

[31]. Kanduma EG, Mwacharo JM, Githaka NW, Kinyanjui PW, Njuguna JN, Kamau LM, et al. Analyses of mitochondrial genes reveal two sympatric but genetically divergent lineages of *Rhipicephalus appendiculatus* in Kenya. Parasit Vectors. 2016;9:353.

[73]. Murrell A, Campbell NJH, Barker SC. Phylogenetic Analyses of the Rhipicephaline Ticks Indicate That the Genus Rhipicephalus Is Paraphyletic. Mol Phylogenet Evol. 2000;16:1-7.

[74]. Beati L, Keirans JE. Analysis of the systematic relationships among ticks of the genera Rhipicephalus and Boophilus (Acari: Ixodidae) based on mitochondrial 12S ribosomal DNA gene sequences and morphological characters. J Parasitol. 2001;87:32-48.
