# Supplementary material for: Mitochondrial phylogeography and population structure of the cattle tick Rhipicephalus appendiculatus in the African Great Lakes region
Source: Parasit Vectors. 2018 May 31;11:329. doi: 10.1186/s13071-018-2904-7 (PMC5984310; doi:10.1186/s13071-018-2904-7)
Supplement: Supplementary file 8 — Figure S2. Neighbor-joining tree of 12S haplotype sequences for R. appendiculatus across African countries. (DOCX 18 kb) [file 13071_2018_2904_MOESM8_ESM.docx]

**Additional file 8: Figure S2.** Neighbor-joining tree of *12S* rRNA haplotype sequences for *R. appendiculatus* across African countries. The evolutionary distances were computed using the Tamura 3-parameter method. Bootstrap values (>60) are displayed above nodes. The values in bracket behind haplotypes names correspond to the frequency of each haplotype. Haplotype sequences (12SH1-9) obtained in the present study are indicated by a black square. *Rhipicephalus eversti* and *R. microplus* obtained in the present study and *R. turanicus* from GenBank (accession number: DQ849231) were used as outgroups
